# Hepatoprotective Plants from Bangladesh: A Biophytochemical Review and Future Prospect

**DOI:** 10.1155/2021/1633231

**Published:** 2021-08-31

**Authors:** Razina Rouf, Puja Ghosh, Md. Raihan Uzzaman, Dipto Kumer Sarker, Fatima Tuz Zahura, Shaikh Jamal Uddin, Ilias Muhammad

**Affiliations:** ^1^Department of Pharmacy, Bangabandhu Sheikh Mujibur Rahman Science and Technology University, Gopalganj 8100, Bangladesh; ^2^Pharmacy Discipline, Life Science School, Khulna University, Khulna 9208, Bangladesh; ^3^National Center for Natural Products Research, School of Pharmacy, Research Institute of Pharmaceutical Sciences, University of Mississippi, University, MS 38677, USA

## Abstract

Liver diseases are quite prevalant in many densely populated countries, including Bangladesh. The liver and its hepatocytes are targeted by virus and microbes, as well as by chemical environmental toxicants, causing wide-spread disruption of metabolic fuctions of the human body, leading to death from end-stage liver diseases. The aim of this review is to systematically explore and record the potential of Bangladeshi ethnopharmacological plants to treat liver diseases with focus on their sources, constituents, and therapeutic uses, including mechanisms of actions (MoA). A literature survey was carried out using Pubmed, Google Scholar, ScienceDirect, and Scopus databases with articles reported until July, 2020. A total of 88 Bangladeshi hepatoprotective plants (BHPs) belonging to 47 families were listed in this review, including Euphorbiaceae, Cucurbitaceae, and Compositae families contained 20% of plants, while herbs were the most cited (51%) and leaves were the most consumed parts (23%) as surveyed. The effect of BHPs against different hepatotoxins was observed via upregulation of antioxidant systems and inhibition of lipid peroxidation which subsequently reduced the elevated liver biomarkers. Different active constituents, including phenolics, curcuminoids, cucurbitanes, terpenoids, fatty acids, carotenoids, and polysaccharides, have been reported from these plants. The hepatoameliorative effect of these constituents was mainly involved in the reduction of hepatic oxidative stress and inflammation through activation of Nrf2/HO-1 and inhibition of NF-*κ*B signaling pathways. In summary, BHPs represent a valuable resource for hepatoprotective lead therapeutics which may offer new alternatives to treat liver diseases.

## 1. Introduction

The liver is one of the vital organs in the human body that is responsible for metabolism, excretion, and regulation of body homeostasis [1]. Therefore, the liver and its hepatocytes are the major targets of various toxicants (i.e., heavy metals, toxins, drugs, and other chemicals), microbes, and viral infections [2]. Harmful effects of the abovementioned factors on the liver and its hepatocytes include inflammatory (i.e., hepatitis) or non-inflammatory (i.e., hepatosis) liver diseases, liver fibrosis (i.e., cirrhosis), jaundice, and alcoholic liver disease. Liver diseases are now considered as one of the major global health problems, particularly in low- and middle-income countries where it gives the highest burden but largely neglected [3]. Liver disease affects millions of people worldwide, with about 2 million dying annually, and the rates of affecting are increasing sharply over the years irrespective of age, sex, region, and race [4, 5]. Liver diseases, including jaundice and chronic viral hepatitis, as well as nonalcoholic fatty liver, are one of the major treatment burdens in Bangladesh. About eight million people are reported to have viral hepatitis, Hep B (HBV) and C (HCV), and also, frequent outbreak of hepatitis A (HAV) and E (HEV) have been reported in Bangladesh [6]. It is estimated that one in five maternal deaths associated with acute jaundice occurred in Bangladesh as well as increasing trend of fatty liver diseases [7]. Drugs that are currently available for the treatment of liver diseases suffer a number of shortcomings including side effects, poor bioavailability, stability, and selectivity [8]; thus, it is necessary to search new drugs with optimum efficacy, stability, selectivity, and safety for the treatment of liver diseases.

Medicinal plants play a key role in human health, and about 80% of the world's population in developing countries relies on the use of plant-based traditional medicine [9]. The use of medicinal plants for the treatment of liver diseases has a long history. Scientific study has increased with ethnopharmacological plants that possess strong hepatoprotective activity [10]. The term “hepatoprotective” means either to protect or prevent the liver damage. A number of scientific studies on such local plants and their herbal formulations around the world have been recorded as hepatoprotective [8, 10–17]. Numerous phytoconstituents have already proved to protect liver diseases in both in vitro and in vivo settings [18–21]. It is obvious that traditional herbal medicines are a natural treasure because of their chemical diversity, affordability, availability, few side effects, and diverse pharmacological activity [8, 22]. It is established that traditional knowledge on the medicinal plants has indispensable importance on new drug discovery [23], which attracted immense interest by the scientists over many decades.

The use of medicinal plants for the treatment of different diseases by Indo-Aryans has been reported in Rig-veda in 4500–1600 BC [24, 25]. Bangladesh, which belongs to the Indian subcontinent, also possesses a rich heritage of herbal medicines [26]. About 500 species of medicinal plants are growing in Bangladesh, and among these, more than 250 species are currently in use for the preparation of herbal medicines using the traditional approach and about 80% of rural population of Bangladesh depends on traditional herbal medicine for their primary healthcare [25].

Ethnobotanical use of Bangladeshi medicinal plants has a long history of use in the treatment of liver diseases including jaundice, ascites, liver cirrhosis, hepatitis, liver enlargement, inflammatory liver, sclerosis of the liver, and other liver diseases [26]. Traditional healers of Bangladesh have strong believed on alternative natural plant-based medicine that has few side effects than modern synthetic medicine. The traditional medicinal practitioner, Kabiraj, have developed a number of herbal and ayurvedic formulations in Bangladesh for the treatment of liver diseases [27]. This is one of the affordable and accessible treatment options in liver diseases by rural people because of low cost and lack of access to modern treatment [7]. It is well known that plant-derived natural molecules including ﬂavonoids, terpenoids, sterols, and antioxidants possess diverse therapeutic effects including hepatoprotective activity [10]. The presence of these bioactive phytoconstituents has made these traditionally used plants highly effective against liver diseases. However, there is no up-to-date report on ethnopharmacological and phytochemical investigations for active constituents of Bangladeshi medicinal plants used to treat liver diseases. The aim of this review is to summarize the potential of active compounds from plants used traditionally for liver diseases, as well as the underlying proposed mechanisms of action by compiling both in vitro and in vivo studies.

## 2. Literature Search Strategy and Data Extraction

Plants that are currently used locally to treat liver diseases are listed in two ethnopharmacological books, namely, the Medicinal Plants of Bangladesh with Chemical Constituents and Uses and the Medicinal Plants of Bangladesh [26, 28]. A comprehensive list of such plants (Table 1) was developed from these two book sources. Furthermore, a complete literature survey on each plants was conducted by PubMed, Scopus, Google Scholar, Web of Sciences, and ScienceDirect databases, using keywords “Bangladeshi medicinal plants” and “plant extracts”, and then refined with the additional keywords “hepatoprotective,” “liver diseases,” “hepatoprotective activity,” and “isolated compound.” In this review, the following surveys were conducted, including (a) in vivo, in vitro, and clinical studies of plant extracts and compounds for liver diseases, (b) studies concerning the concentrations, doses, and route of administration of extracts and/compounds, and (c) studies concerning MoA associated with the hepatoprotective activity of extracts and/or constituents.

## 3. Results and Discussion

### 3.1. Hepatoprotective Plants

**Figure 1:**
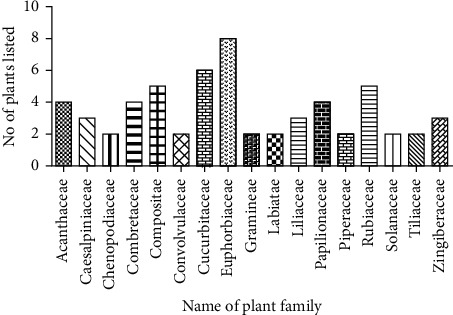
Graphical presentation of the Bangladeshi medicinal plant family containing more than one plant traditionally used to treat liver diseases.

**Figure 2:**
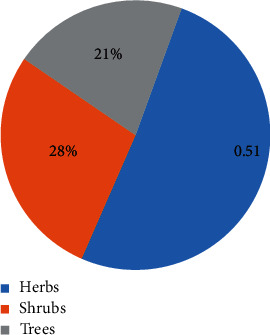
Bangladeshi medicinal plants with their % of habit traditionally used to treat liver disease.

**Figure 3:**
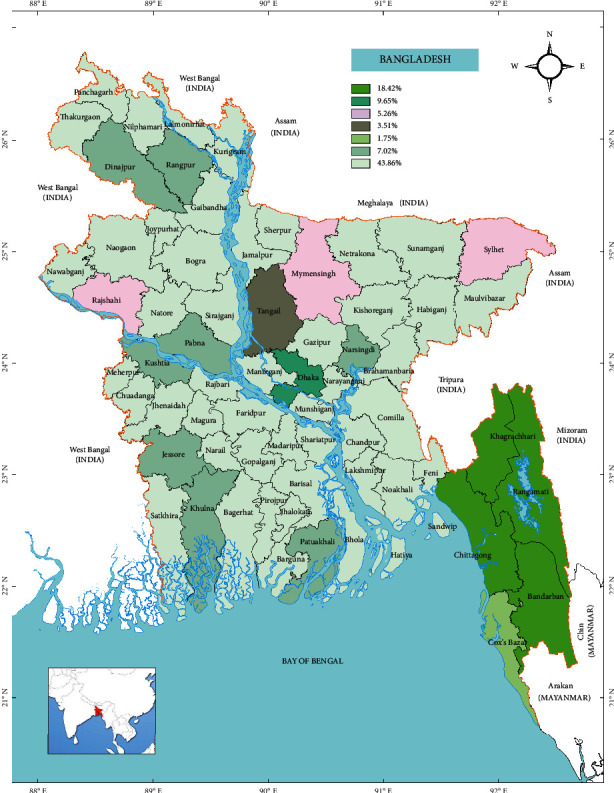
Percent distribution of Bangladeshi hepatoprotective plants listed in Table 1 [26].

In this review, a total of 88 species belonging to 47 families and more than 75 genera were selected based on various liver diseases including jaundice, ascites, liver cirrhosis, hepatitis, liver enlargement, inflammatory liver, and sclerosis of the liver [26, 28]. Table 1 shows the common names, plant parts, dosage form of extracts, and recommended use for liver diseases of these plants, and the most used form of preparation was juice (21%), hydroalcoholic extract (14%), and the decoction or infusion (5–19%). The distribution of various species with respect to families is shown in Figure 1, while the percentage distribution of various categories of samples, such as herbs, shrubs, and trees with 51%, 28%, and 21%, respectively, is shown in Figure 2. Finally, Figure 3 illustrates that about 43% of the total recorded plants are distributed all over the country, including the districts of Chittagong, Dhaka, Mymensing, Sylhet, and Rajshahi.

**Figure 4:**
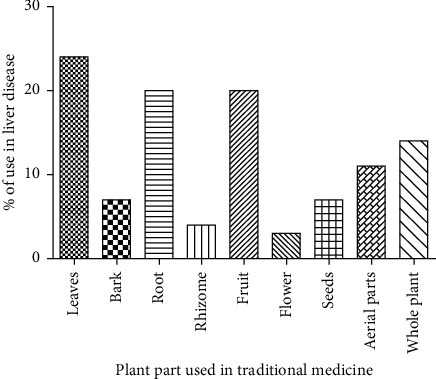
Percentage of plant parts used in treatment of liver disease and jaundice. It is shown that leaves, root, and fruits are the most popular plant parts of Bangladeshi medicinal plants used to treat liver diseases.

Among the plant parts employed for ethnopharmacological use, leaves were highly utilized with 23%, followed by roots, fruits, whole plants/aerial parts, seeds, bark, rhizomes, and flowers (19%, 18%, 12%, 6%, 5%, 3%, and 2%, respectively) (Figure 4).The methods of preparation of each plant parts showed the unique indigenous knowledge of the traditional healers [25, 29]; thus, different methods of preparation carry different active constituents. Here is a summary of active plants from various plant families.

#### 3.1.1. Plants from Euphorbiaceae

**Figure 5:**
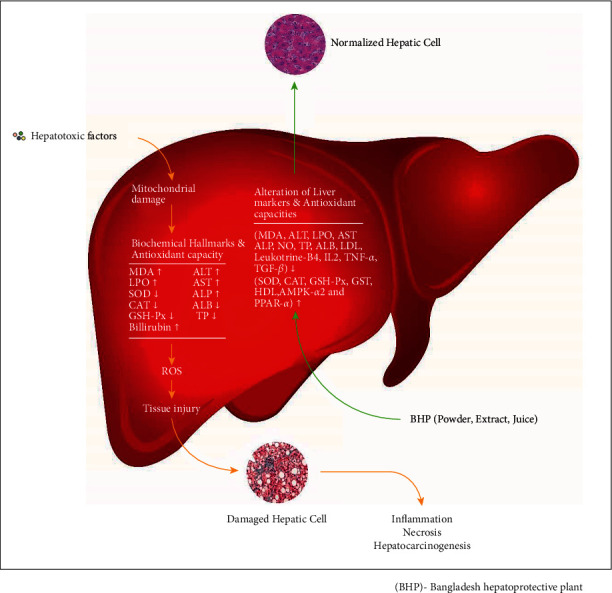
Effects of hepatotoxic chemicals and hepatoprotective plants on liver injury, inflammation, and oxidative stress.

Euphorbiaceae is a large family containing about 300 genera and over 5000 species [30]. The ethnomedicinal significance of this family has a long tradition because of its diverse secondary metabolites [31]. The present review demonstrated that, among the 47 families, Euphorbiaceae possesses eight species, including *Baliospermum montanum, Croton caudatus, Croton oblongifolius, Euphorbia tirucalli, Flacourtia jangomas, Phyllanthus acidus, Phyllanthus emblica*, and *Phyllanthus freternus*, that have been traditionally used to treat liver diseases (Table 1). *B. montanum,* locally known as Dantimul, is a leafy branched undershrub, distributed throughout Bangladesh and India. Decoction of the roots has been used to treat jaundice in Bangladesh [26], as well as in the Ayurvedic system of medicine [32]. A number of in vivo and in vitro hepatoprotective models have been developed for extract or isolated compounds to prevent or cure liver toxicity induced by various hepatotoxins, such as thioacetamide (TAA), carbontetrachloride (CCl_4_), and paracetamol (APAP) [33]. The liver protective effect of alcoholic and aqueous extract of *B. monatum* root was reported by restoring the biochemical changes induced by paracetamol (2 mg/kg) in rat, and the results were comparable to the silymarin (Table 2). The dose-dependent hepatoprotective effects of methanolic fraction and its subfractions in either CCl_4_-, thioacetamide-, or paracetamol-induced liver damage rats were comparable to the protective effect of silymarin at 100 mg/kg, and the protective effect of root extracts was believed to be for their antioxidant effects (Figure 5) [34–37].

#### 3.1.2. Plants from Cucurbitaceae

There are six local species from five genera of the Cucurbitaceae family [38] which have been used to treat liver disease (Table 1). Among these, the fruits of *C. colocynthis* and *C. lanatus* are cross compatible and reported to protect hepatic injury induced by different hepatotoxins (Table 2), and the fruit and root of *C. colocynthis* have been used for the treatment of jaundice [39, 40]. Initial toxicity study of its methanolic extract in mice was found to be safe upto a dose of 2000 mg/kg [40]. A number of in vivo investigations of the crude extract showed dose-dependent hepatoprotective activity induced by hepatotoxins, such as APAP, CCl_4_, cisplatin, or polluted water, in rats (Table 2). The alcoholic extract of *C. colocynthis* fruits showed protective activity against APAP-induced hepatotoxicity in rats at 200–300 mg/kg via decreasing the elevated level of liver enzymes, and the results were comparable to the marketed preparation Liv. 52 (1 mL/kg) and silymarin (100 mg/kg), respectively [40, 41]. Pretreatment with chloroform fractions of this fruits has also been reported to reduce the impact of CCl_4_ and lipopolysaccharide toxicity on the serum liver markers which was comparable to the Chinese drug Bifendate pills (a synthetic intermediate of schisandrin C). The mechanism of the protective effect of *C. colocynthis* might be involved in the reduction of cellular oxidative stress through increasing antioxidant defense systems as well as upregulating the cellular antioxidant enzymes level [40, 42–45].

The juice of *C. lanatus* (local name: tarmuj) fruit pulp possesses antioxidant activity and protects liver damage in ethanol-induced liver toxicity in rat via increasing cellular glutathione (GSH) and catalase (CAT) enzymes, which supports the traditional use to treat liver damage [46]. The dose-dependent liver protective effect of MeOH or EtOH seed extracts (200–400 mg/kg) showed a significant reduction of oxidative stress and improved drug metabolizing enzyme activity in the liver [47–49]. The seed extracts also protect liver fibrosis via inhibiting alpha-smooth muscle actin (*α*-SMA) and transforming growth factor-*β*1 (TGF-*β*1) protein expression in CCl_4_-induced hepatotoxicity in the rat model [47], whereas the EtOH extracts of the leaf of *C. lanatus* ameliorate and reverse damage of the rat liver tissues induced by CCl_4_ via reduced congestion and necrosis as well as normalized serum AST, ALT, ALP, and bilirubin concentrations [50].

*Momordica charantea* has been used for various medicinal values, especially diabetes, which is recommended for jaundice as well (Table 1). In India, the fruit juice or leaf decoction has been used traditionally for hepatitis and jaundice [51, 52]. Mada showed that the extract of *M. charantea* is quite safe and found the LD_50_ was more than 5000 mg/kg [53]. A number of reports showed that the hydroalcoholic or aqueous extract of *M. charantea* leaves dose-dependently (100–400 mg/kg) protect hepatotoxicity induced by CCl_4_ in the rat model in which the extract supplementation restored the elevated level of different liver toxicity markers, and the results were comparable to marketed liver protective preparation silymarin (50 mg/kg) or Liv 52 (5 mL/kg) [53–55]. The aqueous extract of *M. charantea* fruit also reported to protect dose dependently liver toxicity in mice or rats by reducing the elevated liver markers as well as attenuating oxidative stress (Table 2) [51, 56, 57].

#### 3.1.3. Plants from Compositae/Asteraceae

Plants from Compositae (Asteraceae) have been used in traditional medicinal practice in Bangladesh to treat liver diseases for a long time. It is a large family of flowing plants which consists of over 32,000 species within over 1,900 genera and 13 subfamilies [58]. Decoction of different plant parts of local species *Carthamus tinctorius, Eclipta alba, Elephantopus scaber, Sonchus wightianus*, and *Wedelia chinensis* has been used in the treatment of liver diseases, including jaundice and enlargement of the liver (Table 1). The plant *C. tinctorius,* known as safflower, has been used as a hepatotonic in Unani medicine of India and Bangladesh, whereas in Jamaica and the Philippines, the flowers are used for jaundice [59].The MeOH extract of *C. tinctorius* flowers reported to protect CCl_4_-induced liver injury in rat via the antioxidant and anti-inflammatory mechanism, as well as reduced the level of biochemical markers (Table 2) [60]. The seed oil and leaf extract of *C. tinctorius* also possess liver protective activity and showed protective effect via reducing the liver toxicity biomarkers in alloxen-induced diabetic rat or CCl_4_-induced liver toxicity in rat [61, 62].

*E. alba* is an annual herb that is traditionally used in Bangladesh against jaundice and enlargement of the liver [26]. In India, the plant has been used as a tonic and in the treatment of enlargement of the liver [63]. It has also been reported to be used in Ayurvedic medicine to treat infective hepatitis in adults and children [64]. In Chinese medicine, the palnt has use in liver and kidney problems [65]. Sing showed that *E. alba* is safe up to 2 g/kg in mice [64], and later, Lal demonstrated the LD_50_ 5 g/kg [66]. The aqueous extract of *E. alba* leaf showed protection against liver damage induced by either CCl_4_ or ethanol in rat via reduced oxidative stress on the liver by elevating the antioxidant enzyme level (Table 2) [65, 67]. Another study conducted by Lal showed that the MeOH extract of leaves and CHCl_3_ extracts of roots reduced the level of lysosomal enzyme and protected from hepatic damage in CCl_4_-treated rats [66]. The dose-dependent hepatoprotective effect (62.5–500 mg/kg) exhibited by alcoholic aerial part extract of *E. alba* was found to be due to some protection of hepatic drug-metabolizing enzymes, and it restored the impaired excretory capacity of the liver in CCl_4_-treated animals [64].

The crused roots of *E. scaber* are used traditionally to treat liver diseases in Bangladesh [26]. In Malaysia, the plant has been used as a liver tonic, whereas in Brazil, root juice has a common use in treating liver troubles and hepatitis [68]. In China, a herbal drink Yi-GanYin containing *E. scaber* is used to protect the liver against different diseases including liver cancer, cirrhosis, and hepatitis [69]. Teng-Khia-U is another Taiwanease herbal medicine that also contains *E. scaber* with other plants and claimed to possess hepatoprotective and anti-inflammatory activity [70]. The alcoholic extract of the root and leaves of *E. scaber* reported to possess antioxidant activity and reduced the oxidative stress in rat treated with CCl_4_ or ethanol and protect from liver damage dose dependently (Table 2) [68, 70, 71].

#### 3.1.4. Plants from Acanthaceae

*Andrographis paniculata* Burm. (Acanthaceae) is a common and widely used medicinal plant from South-East Asia, including Bangladesh, that has been used to treat liver diseases [26, 72, 73]. The leaves and aerial parts of this plant have also been used in Chinese medicine against liver problems [74]. In the Ayurveda system, about twenty-six different polyherbal preparations containing this plant have been recorded [75]. *A. paniculata* extract has also been reported to be effective in ameliorating the chronic hepatitis B virus infection [76]. A number of animal studies were conducted with ethanolic or aqueous extract of leaves of *A. paniculata* supplementation against different hepatotoxin (TAA, APAP, CCl_4_, and hexachlorocyclohexane (BHC))-induced liver toxicity in the rat or mice model, and the results demonstrated that supplementation of *A. paniculata* normalized cellular oxidative stress and dose-dependently protected against liver toxicity as assessed in terms of either reduced serum marker enzymes, as well as restored the liver tissues antioxidant enzymes (Table 2) [77–80]. *A. paniculata* extract at a dose of 50–200 mg/kg could protect the liver by restoring antioxidant enzymes as well as reduction (appx. 33–48%) of lipid peroxidation in the liver [81]. The plant extract contains different phenolic phytoconstituents and believed to act as antioxidants as a part of its mechanism of lipid peroxidation [82, 83]. The water extract of the plant exhibited greater antioxidant activity than ethanolic extract because of high phenolic contents in water extract than ethanolic extract [84, 85].

#### 3.1.5. Plants from Combretaceae

A number of species of the genus *Terminalia* belonging to the Combretaceae family have been used to treat liver problems in the traditional medicinal practice of Bangladesh. *Terminalia arjuna, Terminalia chebula, and Terminalia belerica* are three important medicinal plants that have been used in Ayurvedic medicine for over centuries, primarily as a tonic for the heart and liver [86, 87]. The use of aqueous bark and ethanolic fruit extracts of *T. arjuna* and *T. chebula* showed liver-protective activity in antitubercular drug (single INZ or in combination of RIF, INZ, and pyrazinamide (PZA))-induced liver toxicity in rats at a dose of 50–200 mg/kg (Table 2). The hepatoprotective effect of these extracts was due to their prominent antioxidative and membrane-stabilizing activities [88, 89]. Pretreatment of rats with the extract of *T. arjuna* bark and *T. chebula* fruit also showed the hepatoprotective potential against paracetamol-/CCl_4_-induced liver damage through a significant reduction of serum liver marker enzymes, which was comparable to silymarin [90–92]. The hepatoprotective activity of *T. belerica* fruit extract was observed by shortened hexobarbitone sleeping time and zoxazolamine paralysis time by inducing drug-metabolizing enzyme and dose-dependent elevation of serum transaminases and bilirubin in CCl_4_-induced liver injury rats [93].

#### 3.1.6. Plants from Papilionaceae

*Cajanus cajan* (Papilionaceae) is another popular Bangladeshi plant which has been used in local traditional medicinal practice to treat liver diseases [26]. The juice of leaves is used to treat jaundice by the folklore practitioners of Bangladesh and India [26, 52]. A number of investigations were conducted to evaluate *C. cajan* extract for its liver-protective activity (Table 2). Pretreatment of alcoholic extract of *C. cajan* leaves or aerial parts showed liver-protective activity via the reduction of elevated liver enzymes in CCl_4_- or APAP-induced liver injury rats [94, 95], as well as antiperoxidative activity by induction of the antioxidant enzymes, namely, CAT, SOD, GPx, and GST, in hepatitis rats induced by GNH_2_ [96]. The methanolic aqueous fraction of *C. cajan* leaves protects from hepatocyte in alcohol-induced liver-damaged rat through normalized UDP-glucuronosyl transferase (UGT) activity and upregulates the expression of UGT-2B with nuclear translocation of nuclear factor erythroid-2-related factor-2 (Nrf2) [97].

#### 3.1.7. Plants from Caesalpiniaceae

The plants *Cassia fistula* and *Caesalpinia bonducella* belonging to the family Caesalpiniaceae have been used in the treatment of liver diseases, and a number of reports have been published on their different parts that possess liver-protective activity (Table 2). In vivo data showed that the liver-protective effect by different extracts of *C. fistula* were observed at doses 200–800 mg/kg (b.w) via decreasing various elevated liver biomarkers in chemical-induced liver toxicity in animals (Table 2) [98–102]. Drug-induced hepatotoxicity was also prevented by the ethanolic extract of leaves of *C. fistula* by reduced oxidative stress and other liver biomarkers in diethyl nitrosamine or INZ with RIF-induced liver toxicity in rats [103, 104], whereas the alcoholic or AQ extract of leaves of C. *bonducella* showed hepatoprotective activity observed by decreasing the activity of serum liver biomarkers [105–107] as well as reduced oxidative stress via increasing the levels of antioxidant enzymes [108, 109].

#### 3.1.8. Plants from Liliaceae

*Aloe barbadensis* (*Aloe vera*) and *Asparagus racemosus* are two popular ethnomedicinal plants belonging to the Liliaceae family that have been used to treat liver diseases. Different hepatotoxin-induced liver toxicities in rat models were used to evaluate their liver-protective activity (Table 2). Among these, the fresh AQ extract of *A. barbadensis* has been studied for its hepatoprotective activity in a various animal models, and the results confirmed its protective activity that could play a therapeutic role against in either CCl_4_, APAP, alcohol, or drugs (lindane, INZ, or RIF)-induced liver damage by improving liver enzyme activities and improved antioxidant enzymes in intoxicated rat liver tissue [110–114].

A Korean pharmaceutical preparation of *A. barbadensis* called ACTIValoe®N-931 (mixture of *A. barbadensis* and *Silybum marianum* extracts) has been studied interperitoneally in the CCl_4_-induced hepatotoxicity and liver fibrosis rat model, and the results demonstrated that both acute hepatotoxicity and liver fibrosis were prevented via restoring the serum aminotransferase, lipid peroxidation, and increasing the hepatic glutathione content. The ACTIValoe®N-931 also attenuated the increase in TNF-*α* and induced nitric oxide synthase (iNOS), cyclooxygenase-2 (COX-2), and mRNA expressions in acute hepatotoxicity induced by CCl_4_ in rats [115]. Besides the in vivo assays, a clinical study was also conducted using the fresh juice of *A. barbadensis* among male and female patients of age 15–65 years diagnosed clinically and biochemically with acute viral hepatitis. A number of liver biochemical markers were measured at the end of 2, 4, and 6 weeks, and it was found that the juice extract of the plant has liver-protective activity compared to the control group, which confirmed the traditional uses of *A. barbadensis* for the treatment of liver diseases [116].

#### 3.1.9. Plants from Zingiberaceae

*Curcuma longa* (turmeric; Zingiberaceae) has been used as a herbal medicine owing to its multipharmacological activities [117, 118]. It has been used locally in traditional medicine for the treatment of liver diseases (Table 2). Liver cirrhosis induced by TAA in rats was prevented by the ethanolic extract of turmeric through the inhibition of oxidative stress markers and upregulation of liver antioxidant enzymes and the anti-inflammatory mechanism which restored the elevated cytokines TGF-*β*1 and TNF-*α*, as well as enhanced apoptosis of damaged hepatocytes as a protective mechanism and downregulated inflammatory effects and fibrogenesis of the liver [119]. Liver-protective activity through upregulation of hepatic antioxidant enzymes and restoring of various liver biomarkers by the crude extract of *C. longa* were also supported by other studies of this plant with similar activity in different hepatotoxin (CCl_4_, GNH_2_, or tubercular drug)-induced liver damage in the animal model [120–122]. Other reports also showed that alcoholic extract of turmeric alleviate of hepatotoxic effects caused by HgCl_2_ in rats through a protective effect on drug metabolizing CYP-2E1 enzymes, viz., aniline hydroxylase (AH) and amidopyrine-N-demethylase (AND) [123]. The extract of turmeric also reported to improve liver activity via enhancing its antioxidant activity in alloxan-induced diabetic rat liver toxicity [124].

#### 3.1.10. Plants from Other Families

A number of other traditional plants belonging to various families also have been used in the management of liver diseases. *Amaranthus spinosus* (Acanthaceae) is a leafy vegetable that has been used to treat jaundice. A number of studies found on its extracts of whole plant showed dose-dependent (100–400 mg/kg) liver-protective activity against various hepatotoxin (CCl_4_, GNH_2_, and APAP)-induced liver toxicity in rats via induction of liver antioxidant enzymes and inhibiting oxidant enzyme MDA, as well as restored the elevated level of serum liver biomarkers and cellular architecture [125–127]. The latex and extract of *Argenome mexicana* (Papaveraceae) have been reported to be used in the herbal medicine to treat jaundice in Bangladesh. Literature study found that the supplementation of leaf extract or dietary leaves of *A. mexicana* has the ability to reduce the activities of liver marker enzymes and protect against liver injury induced by CCl_4_ in rat models [128–130]. Similarly, *Bixa orellana* (Bixaceae) also reported to possess inhibition of elevated liver biomarker enzymes activities of CCl_4_- or EtOH-included hepatotoxic rats to protect against liver injury (Table 2) [95, 131–133].

The hepatoprotective activity of various parts of *Boerhavia diffusa* (Nyctaginaceae) has been well studied in different hepatotoxin (TAA, CCl_4_, EtOH, and IB)-induced rat liver toxicity (Table 2). The MeOH extract of *B. diffusa* root restored the elevated liver markers and reduced oxidative stress, as well as normalized liver histological changes, to protect against hepatotoxicity in ibuprofen- (IB-) induced liver toxicity in rats [134]. The alcoholic or hydroalcoholic extract of *B. diffusa* whole plant showed protective activity in CCl_4_-induced liver injury rat via the protection of drug-metabolizing enzymes and restored the elevated liver biomarkers, as well as increased bile flow of the liver [135, 136].

*Carica papaya* (Carieaceae) is another common species that has been used extensively in the liver problem, especially its fruit, either as cooking of unripe fruit or raw eating of ripe fruit [26]. The AQ extract of *C. papaya* leaves and unripe fruit at an oral dose of 100–300 mg/kg showed upregulation of antioxidant enzyme activities in liver tissues and decreased serum liver markers, as well as decreased reduced lipid peroxidation as a protective mechanism in CCl_4_- or APAP-induced hepatotoxicity in rats [137]. The AQ extract of ripe/unripe seeds or dried fruit or MeOH extract of stalk of *C. papaya* also has liver-protective potential that can hamper the activity of liver biomarker enzymes in CCl_4_-induced hepatotoxic rats (Table 2) [138–141]. *Daucas carota* (carrot; Umbelliferae) is another common functional food that has medicinal uses against liver disease. The AQ extract of tuber of *D. carota* showed protective activity against LD-, APAP-, INZ-, or EtOH-induced liver toxicity in rats through altering lipid profile, restored depressed antioxidant systems, and decreased levels of serum enzymes (Table 2) [142, 143]. Similar activity was also found for the oil extract and its phenolic rich fraction in CCl_4_-induced hepatic injury rats where the oil extract showed hepatoprotective activity via reduction of cellular oxidative stress and restored the elevated levels liver markers [144].

### 3.2. Antioxidative Plants for Hepatoprotection

**Figure 6:**
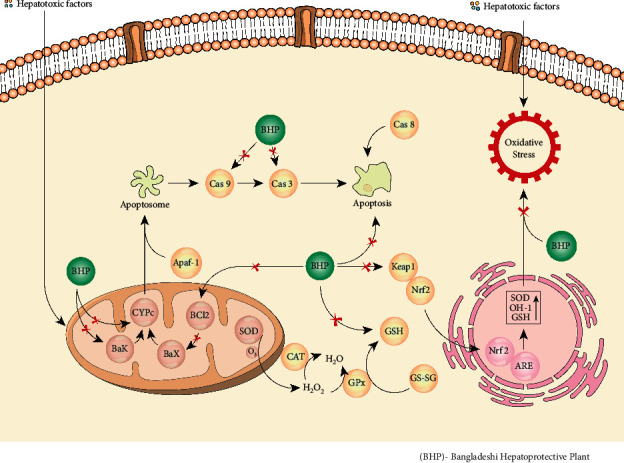
Proposed molecular mechanism of hepatoprotective activity.

Hepatotoxicity or hepatic injury or liver damage occurs mainly through oxidative stress, inflammation, or lipid peroxidation which ultimately inhibits liver regeneration, mitochondrial damage, and finally, cell death [145]. As a consequence, a number of biochemical marker alterations and upregulatin of cellular antioxidative defense mechanisms occurred as a reflection of hamper of liver function (Figure 5) [145]. Treatment with antioxidant can prevent and cure liver diseases by balancing oxidative stress. It is reported that antioxidants can enhance dissociation of Nrf2 from the complex by either modifying kelch-like ECH-associated protein-1 (Keap1) or Nrf2 phosphorylation which causes activation of Nrf2 (Figure 6). The activated Nrf2 translocates into the nucleus, binds to antioxidant response element (ARE), and upregulates the gene expression of antioxidant enzymes and phase II detoxifying enzymes, which protects and cures cellular damage [146]. A number of reports also showed that some antioxidants or antioxidant-rich plant extracts protect against hepatotoxin-induced liver damages by upregulation of activation of Nrf2 [147–149].

A number of other indigenous ethnomedicinal plants belonging to different families have also reported its hepatoprotective activity through their antioxidant mechanism (Table 2). However, a number of plants have been used in traditional medicine to treat liver diseases, although no report was found on their selective hepatoprotective activity, including *Aloe indica*, *Allamanda* cathartica*, Anagallis arvensis, Borassus flabellifer, Caesalpinia pulcherrima, Corchorus capsularis, Corchorus olitorius, Croton caudatus, Croton oblongifolius, Flacourtia jangomas, Ipomoea aquatica* Forsk.*, Justicia gendarass* Burm.*, Lagenaria siceraria, Mussaenda glabrata, Meyna spinosa, Phyllanthus freternus, Piper longum, Pavetta indica, Saccharum officinarum, Sonchus wightianus, Semecarpus anacardium, and Tamarix troupiihole* (Table 1). Therefore, these Bangladeshi medicinal plants could be a promising source to explore further to evaluate their liver protective activity and further identify the active principle.

### 3.3. Hepatoprotective Phytoconstituents

Natural product small molecules (NPSMs) or active fractions containing NPSMs that possess different pharmacological actions, especially antioxidant and anti-inflammatory activity, have recently attracted potential attention to treat liver diseases [150]. A variety of plants and fruits have been used to protect liver function that possess different phytoconstituents including phenolics, flavonoids, coumarins, alkaloids, essential oils, glycosides, xanthenes, carotenoids, organic acids, lignins, and monoterpenes [151]. A number of hepatoprotective phytoconstituents belonging to different chemical classes have been isolated and reported so far from these Bangladeshi plants (Table 3). The various active constituents isolated from these plants were assembled based on the class of compounds as follows.

#### 3.3.1. Flavoinoid and Phenolic Compounds

Flavonoid and phenolic compounds occur ubiquitously in plants and are well-known antioxidant and anti-inflammatory compounds [152]. Several investigations on natural antioxidant, especially flavonoids and phenolics from plants, showed a potential effect in different diseases caused by oxidative stress including liver diseases [153]. A number of previous studies demonstrated that these flavonoids can prevent and cure hepatotoxin-induced liver injury in rodents [154–156].

**Figure 7:**
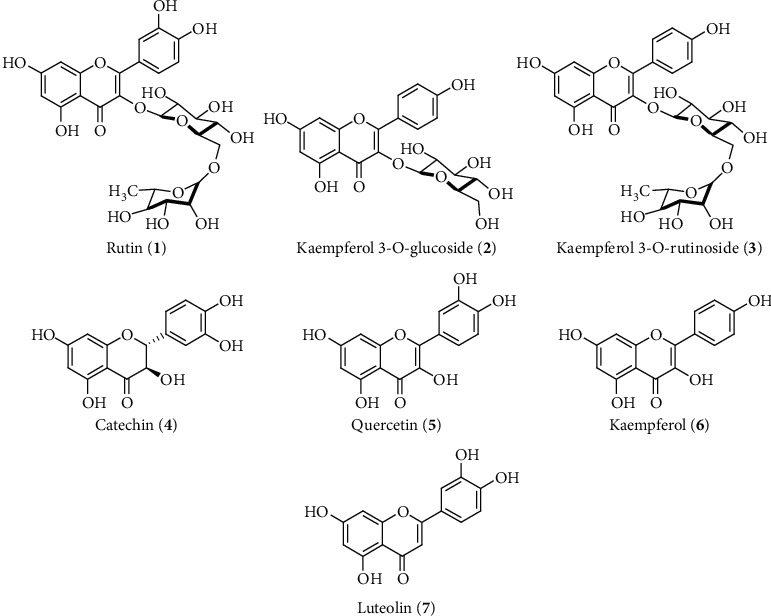
Common hepatoprotective flavonoids identified and isolated from *A. spinosus*, *C. tinctorius, C. fistula,* and *T. purpurea*.

The hepatoprotective plants A. *spinosus*, *C. tinctorius, C. fistula, S. jambos, and T. purpurea* possess flavonoids as a major constituent, and a number of reports showed the hepatoprotective activity of these plants due to their active flavonoids (Table 3). Rutin (**1**), kaempferol 3-O-rutinoside (**2**) or -glucoside (**3**) (336–672 *μ*M/kg), and catechin (**4**) (69 *μ*M/kg) flavonoids were isolated from *A. spinosus*, *C. tinctorius, and C. fistula*, respectively, that exhibited hepatoprotective activity in CCl_4_- or STZ-induced rat liver toxicity (Table 3 and Figure 7). The study revealed that these flavonoids significantly upregulate enzymatic antioxidant systems and regeneration of hepatocytes and, as a result, reduced the elevated serum liver biomarker suggesting their liver-protective effect (Figure 5) [157–159]. Although these studies did not highlight any molecular mechanism of their liver-protective activity, it is well reported that the hepatoprotective activity of these flavonoids might be due to their free-radical-scavenging activity with their anti-inflammatory and antifibrotic responses as well as induction of the Nrf2 signaling pathway (Figure 6) [155, 160, 161].

**Figure 8:**
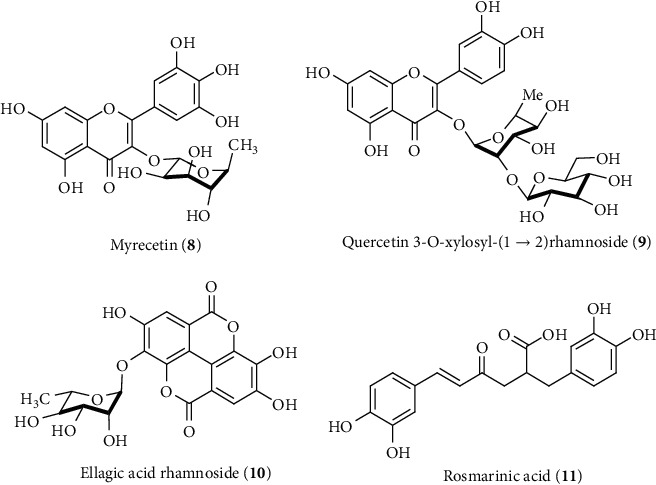
Hepatoprotective flavonoids and phenolics identified from *S. jambos*.

The flavonoid-rich fraction of another hepatoprotective plant (HP) *T. purpurea* containing quercetin (**5**), coumarins, flavonoids, and flavanones protects against rat hepatotoxicity induced by CCl_4_ at a dose of 100 mg/kg dose via reduction of the elevated level of serum SGOT, SGPT, ALP, and bilirubin (Figure 5) [162]. The rutin (**1**), catechin (**4**), quercetin (**5**), kamferol (**6**), and luteolin (**7**) flavonoids have also been isolated from different crude extracts of *A. spinosus*, *C. tinctorius, C. fistula,* and *T. purpurea,* which further supports their reported and traditional liver-protective activity (Figure 7) [163–166]. Flavonoids and phenolics such as myricetin (**8**), quercetin 3-O-xylosyl-(1 ⟶ 2) rhamnoside (**9**), ellagic acid rahmnoside (**10**), and rosmarinic acid (**11**) from *S. jambos* leaf extracts have been identified using HPLC-PDA-MS/MS that showed promising liver-protective activity in CCl_4_-induced liver injury rats (Figure 8) [167]. The extract at a dose of 200 mg/kg reduced the levels of liver markers and increased antioxidant enzymes GSH and SOD. Furthermore, in vitro assay confirmed that pretreatment with the extract inhibited ROS production via prevention of p38 and its target MAPKAP kinase-2- (MAPKAPK-2-) activated signaling cascade in sodium arsenite-induced oxidative stress of HepG2 hepatocytes [167]. The p38 and MAPKAPK-2 are mitogen-activated protein kinase (MAPK) family proteins that regulate the production of inflammatory cytokines as well as play a vital role in hepatoprotective function by restricting ROS accumulation in the liver during oxidative stress [168]. Interestingly, natural flavonoids have already showed their liver-protective activity against oxidative stress via the MAPK signaling pathway [169].

**Figure 9:**
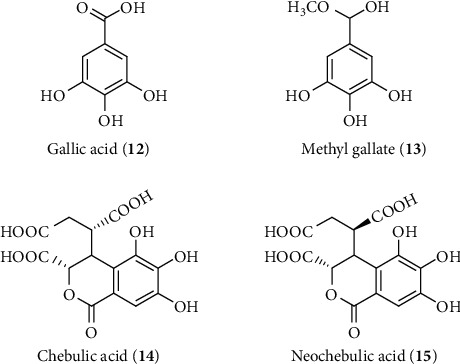
Hepatoprotective phenolics isolated from *L. inermis* and *T. chebula*.

Gallic acid (**12**) and its derivative methyl gallate (**13**) are the common plant phenolics that have been isolated from the BHP of *Lawsonia inermis* and showed significant hepatoprotective effect at an IP (intraperitoneal) dose of 294 *μ*M/kg gallic acid (GA) in CCl_4_-intoxicated rats (Figure 9) [170]. The study did not reveal any molecular mechanism of GA but demonstrated that the protective effect was observed by lowered serum biochemical parameters, a significant reduction of hepatic ROS, and an increase in antioxidant enzymes, as well as normalized hepatocellular necrosis, vacuolization, and inflammatory cell infiltration. Another report also demonstrated that GA has the ability to protect against liver toxicity by enhancing enzymatic antioxidant systems and reduce hepatic inflammation via inducing Nrf2-mediated antioxidant enzymes and attenuating the inflammatory mediators COX-2 through the NF-*κ*B inhibition pathway [171]. Another plant hepatoprotective phenol, chebulic acid (**14**), was isolated (as a mixture with neochebulic acid (**15**)) from *Terminilia chebula* that showed reduction of tert-butyl hydroperoxide- (t-BHP-) induced ROS and cell cytotoxicity and the ratio of GSSH with GSH in isolated rat hepatocytes in vitro at a dose of 280 *µ*M/mL (Figure 9) [172]. A recent in vitro study conducted by Jung et al. confirmed that chebulic acid can dose dependently (0.4, 2 and 2 *µ*M) enhance phosphorylation of MAPK and protect hepatocytes against t-BHP-induced oxidative stress via controlling the activation of Nrf2 and its related cytoprotective enzymes including HO-1 and gamma-glutamate cysteine ligase (*γ*-GCL) [173].

#### 3.3.2. Terpenoids

Among the terpenoid class of NPSMs, a number of diterpene type of compounds have been isolated from BHP that showed potential hepatoprotective activity (Table 3). The diterpene lactone, andrographolide (**16**), is a well-known natural molecule isolated from *A. paniculata* Nees. (Kalmegh) that has been used as a key ingredient in a variety of polyherbal formulations to treat hepatitis, hepatic dysfunction, and hepatic regeneration, as well as a liver tonic, in Bangladesh and the Indian subcontinent [174]. Literature study demonstrated that andrographolide isolated from *A. paniculata* showed liver protection against alcoholic (177–1427 *µ*M/kg, ip)/nonalcoholic (143 *µ*M/kg, p.o) fatty liver or APAP (2–34 *µ*M/kg, p.o)/CCl4 (286 *µ*M/kg, ip)-induced hepatotoxicity (Table 3).

**Figure 10:**
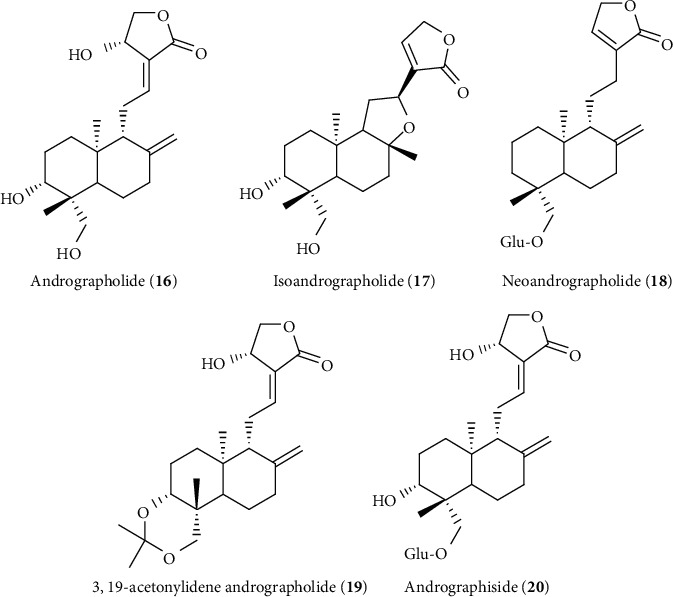
Hepatoprotective diterpene lactone isolated from *A. paniculata*.

The hepatoprotective activity of andrographolide observed via liver regeneration prevents degradation/necrosis of liver cells, upregulates antioxidant enzymes, and inhibits lipid peroxidation [175–178]. Improvement of hepatic biliary function and insulin secretion in hepatocytes has an impact on liver regeneration, prevention of degradation of hepatocytes, or hepatic dysfunction [179, 180]. Interestingly, the protective effect of andrographolide via liver regeneration or prevention of necrosis of liver cells has a close relation with its choleretic effect as well as stimulation of insulin secretion in hepatocytes [181, 182]. It is also reported that andrographolide normalized the hepatic fatty changes, multifocal mononuclear cell infiltration, and hepatocyte ballooning in high-fat-diet fatty liver as a function of its protective effect [183]. Andrographolide derivatives including isoandrographolide (**17**), neoandrographolide (**18**), 3,19-acetonylidene andrographolide (**19**), and andrographiside (**20**) have also been reported to possesses liver-protective effects as andrographolide and even sometimes more potent than andrographolide (Figure 10). Another hepatoprotective study confirmed that the glucoside group with andrographolide (i.e., andrographiside (**20**)) might act as a strong antioxidant than andrographolide itself or neoandrographolide in which andographiside significantly inhibit lipid peroxidation, GSH depletion, and enzymatic leakage of SGPT and ALP compared to andrographolide and neoandrographolide alone [177].

**Figure 11:**
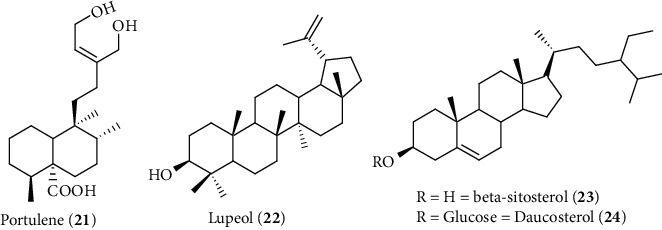
Hepatoprotective phytoconstituents isolated from *P. oleracea* L.

Another new diterpene named portulene (**21**) along with known compounds lupeol (**22**), *β*-sitosterol (**23**), and daucosterol (**24**) has been isolated from the extract of *Portulaca oleracea* that showed a liver-protective effect at a dose 10–50 mg/kg against CCl_4_-induced hepatic injury in rats via the inhibition of leakage of liver enzymes and biomarkers (Figure 11) [184]. The hepatoprotective activity of these phytoconstituents is supported by a previous study of lupeol and *β*-sitosterol that showed liver-protective activity via antioxidant and anti-inflammatory mechanisms [185, 186].

**Figure 12:**
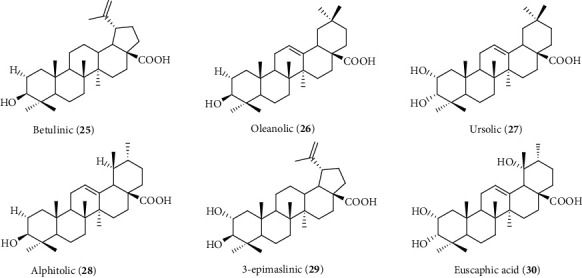
Hepatoprotective triterpene acids isolated from *O. basilicum*.

A number of triterpene acids, namely, betulinic (**25**), oleanolic (**26**), ursolic (**27**), alphitolic (**28**), 3-epimaslinic (**29**), and euscaphic acid (**30**), have been isolated from triterpene-rich CH_2_Cl_2_ hairy root extract of *Ocumum basilicum* L. that showed hepatoprotective activity in CCl_4_-induced hepatotoxicity in experimental animals (Figure 12) [187]. The isolated triterpene acids also dose-dependently (0.1–5 mg/mL) ameliorate liver oxidative stress by inhibition of lipid peroxidation in iron/ascorbate-induced lipid peroxidation in liver homogenate [187]. Interestingly, it is reported that oleanane- and ursane-type triterpenoids are the two largest groups of phytoconstituents that possess noticeable hepatoprotective activities including oleanolic acid and ursolic acid which have been used to treat liver diseases for years in China [188]. The protective effect of oleanolic acid (**26**) against acute liver injury involved its anti-inflammatory activity thorough the activation of peroxisome proliferator-activated receptor alpha (PPAR*α*) and downregulation of the c-Jun NH_2_-terminal kinase (JNK) signaling pathway [189]. The antioxidant effect of ursolic acid (**27**) in the prevention of liver injury involved the modulation of MAPKs and the NF-*κ*B signaling pathway [190]. On the other hand, betulinic acid (**25**) has the ability to prevent hepatic inflammation and fibrosis via the suppression of the TLR4/MyD88/NF-*κ*B signaling pathway [191].

Hepatitis virus (HBV, HAV, and HCV) causes a severe and frequently transmittable disease of the liver, and among these, HBV was the most common one that infected millions of people worldwide. The extract of *O. basilicum* L. was reported to be active against viral hepatitis (HAV) [192]. There was no confirmation about the active principle responsible for the antihepatitis activity of *O. basilicum* L.; however, the triterpene acids, especially betulinic acid, ursolic acid, and oleanolic acid (Figure 12), have been reported to be active against viral hepatitis. The betulinic acid protects mice liver by inhibiting HBV replication in hepatocytes of HBV‐transgenic mice through downregulation of SOD-2 expression as well as inhibition of ROS production and mitochondrial dysfunction [193]. Betulinic acid also inhibits HCV replication in cultured cells, and the molecular mechanism reported that it might downregulate HCV-induced COX-2 expression through the inhibition of phosphorylation of NF-*κ*B and ERK1/2 of the MAPK signaling pathway [194]. The antihepatitis potential of ursolic acid and oleanolic acid was also reported against HBV and HCV viruses. The anti-HBV activity of ursolic acid might be involved in blocking the pathological effects of HBV which confirmed by the study in which ursolic acid reduced the migratory process and matrix metalloproteinase-3 secretion in HBV-X protein-transactivated cell lineages [195], while the anti-HCV activity of oleanolic acid and ursolic acid was observed via inhibition of of viral NS5B RNA-dependent RNA polymerase (RdRp) activity, an enzyme responsible for HCV RNA replication [196].

**Figure 13:**
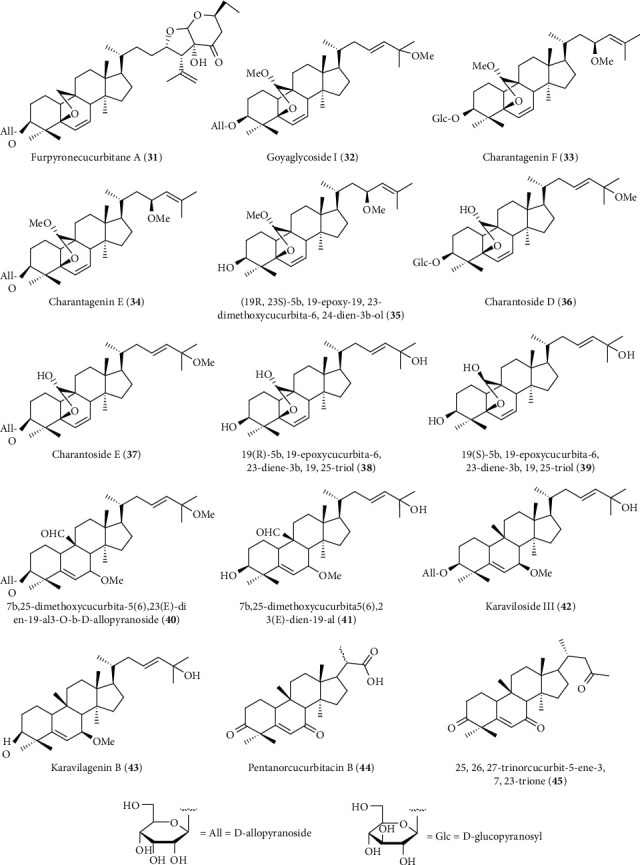
Hepatoprotective cucurbitane-type triterpenoids isolated from *M. charantea.*

Cucurbitane-type triterpene glycosides are another class of hepatoprotective triterpenoids found in *Momordica charantea* L. which has been used as a popular vegetable and traditional medicine to treat liver diseases. A recent study isolated a number of cucurbitane-type triterpene glycosides from the fruits of M*. charantea* including three new furpyronecucurbitane A (**31**), goyaglycoside I (**32**), and charantagenin F (**33**) along with ten known cucurbitane (**34**–**43**) (Figure 13) [197]. All the isolated compounds were evaluated for antihepatic fibrosis activity against murine hepatic stellate cells (t-HSC/Cl-6) and antihepatoma activity against liver cancer cell lines (HepG2 and Hep3B), and karaviloside III (**41**) was found as the most potent molecule with an IC_50_ 3.74–17 *μ*M [197]. Previously, two norcucurbitane-type triterpenoids named pentanorcucurbitacin B (**44**) and 25,26,27-trinorcucurbit-5-ene-3, 7, 23-trione (**45**) were isolated from the same plant that showed cytoprotective potential against t-BHP-induced injury on HepG2 cells with IC_50_ 5–10 mM and was comparable to silybin (Figure 13) [198].

#### 3.3.3. Curcuminoids

**Figure 14:**
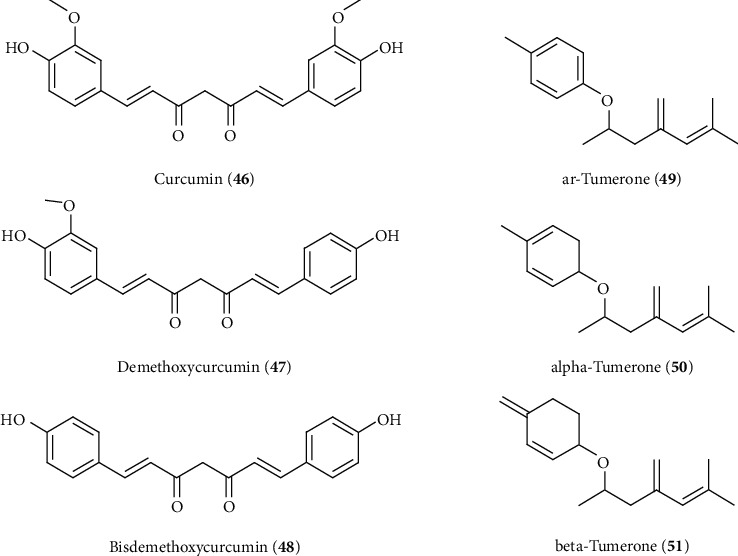
Hepatoprotective curcuminoids and sesquiterpenes isolated from *C. longa*.

Curcuminoids are diarylheptanoids which belongs to natural phenolic compounds, and curcumin (**46**) (60–70%), demethoxycurcumin (20–27%) (**47**), and bisdemethoxycurcumin (10–15%) (**48**) are the major curcuminoids present in turmeric *C. longa* (Figure 14) [199]. Curcumin (**46**), chemically known as (1*E*-6 *E*)-1,7-bis (4-hydroxy-3-methoxy phenyl)-1,6-heptadiene-3,5-dione, is the major bioactive compound isolated from turmeric. Curcumin reported to possess various pharmacological actions including hepatoprotective and antioxidant properties [200]. The hepatoprotective effect of curcumin has been well established via a number of in vitro and in vivo investigations (Table 3). The hepatoprotective activity of curcumin was due to its multitarget function. Curcumin (136–544 *µ*M/kg. p.o) ameliorated liver injury in animals induced by APAP/CCl_4_ or lindane through upregulation of the antioxidant defense mechanism and restored the elevated liver markers via inhibition of hepatic cell degradation and leakage and inhibition of lipid peroxidation [201–203]. Alfatoxin B1-induced hepatotoxicity involved LPO and oxidative DNA damage of liver cells. The antioxidant potential of curcumin protected against aflatoxin B1-induced liver toxicity by restoring the elevated levels of serum marker enzymes and LPO and elevating the antioxidant enzyme levels as well as reduced excretion of DNA adducts [204, 205]. The molecular mechanism of hepatoprotection of curcumin was believed to link with reduction of oxidative stress via the antioxidant activity and activation of the Nrf2/Keap1/ARE pathway and its related phase II detoxifying/antioxidant enzymes including HO-1 and NAD(P)H:quinone oxidoreductase-1 (NQO 1) (Figure 6) [206, 207]. Moreover, curcumin protects CYP 2E1 enzymatic activity against mercuric chloride- (HgCl_2_-) induced hepatotoxicity and oxidative stress in rats [123], which is supported by a previous study of curcumin that it induces peroxiredoxin-6 (Prx-6) and downregulates CYP2E1 as well as Prx1 expression in diet-induced oxidative stress [208]. It is believed that cross regulation of Prx1 and Prx6 is likely to participate in cellular defense against the development of hepatitis. The anti-inflammatory responses of curcumin that protected liver fibrosis induced by dimethylnitrosamine (DMN) were observed along with the reduction of electrical conductivity and leaking of liver biomarkers [209]. Inhibition of the hepatic NF-*κ*B signaling pathway is reported to be a potential pathway to attenuate the inflammatory process in the liver, and a number of investigations confirmed the downregulatory property of curcumin to hepatic expression of NF-*κ*B and its downstream targets [210, 211]. Other reports also showed that curcumin protects against hepatic fibrogenesis through the inhibition of the expression of toll-like receptor 2 and 4 (TLR2 and TLR4) and their ligand molecule high-mobility group protein box-1 (HMGB1) in CCl_4_-indcued rat hepatic fibrogenesis [212]. Interestingly, all TLR signaling pathways have a close relation with NF-*κ*B activation which regulate the expression of inflammatory cytokine genes [213]. Furthermore, curcumin could ameliorate hepatic inflammation and fibrosis by enhancing the degradation of damaged hepatic cells via apoptosis through the inhibition of the expression of proapoptotic genes Bax, Bcl-2 mRNA, and caspase-3 as well as inducing antiapoptotic genes Bcl-xL and upregulating p53 protein expression in APAP- or TAA-induced hepatotoxicity (Figure 6) [214–216].

Curcumin was also a potential natural hepatoprotectve molecule that is effective in viral hepatitis and proved to be active as a host-targeted therapy for HBV infection. Mouler et al. showed that curcumin protects HepG2215 cells from HBV infection via the inhibition of HBV gene expression and replication. The molecular mechanism of the inhibition of replication involved downregulation of peroxisome proliferator-activated receptor-gamma coactivator-1alpha (PGC-1*α*), which is a starvation-induced protein that has a role in the initiation of the gluconeogenesis cascade and may robustly coactivate HBV transcription [217].

#### 3.3.4. Aromatic Compounds

A study reported that *C. longa* yielded aromatic compounds ar-turmerone (**49**) and its derivatives *α*-tumerone (**50**) and *β*-tumerone (**51**) which showed liver-protective activity (0.5% with diet) against d-galactosamine-induced liver toxicity in rats via suppressing the increase of LDB, ALT, and AST levels (Figure 14) [218]. However, the liver-protective mechanism of sesquiterpenes in turmeric was not clear and might be different from that of curcuminoids [219].

**Figure 15:**
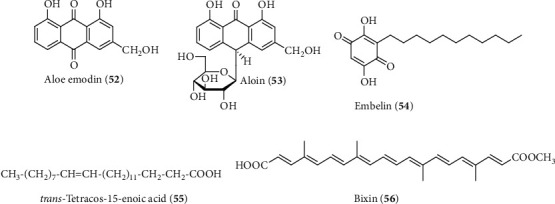
Hepatoprotective compounds isolated from Bangladeshi plants. (a) Anthraquinone derivative aloe emodin and aloin from *A. vera*; (b) para-benzoquinone derivative embelin from of *E. ribes*; (c) fatty acid TCA from of *I. tinctora*; and (d) carotenoid derivative bixin from *B. orellana*.

Aloe emodin (**52**), chemically known as 1,8-dihydroxy-3-hydroxyl-methylanthraquinone, is an anthraquinone derivative and one of the main bioactive components of *Aloe vera* (Figure 15). Literature study demonstrated that the anthraquinone derivative aloe emodin possesses hepatoprotective potential both in vivo and in vitro. Arosio et al. showed that pretreatment of aloe emodin (185 *µ*M/kg, i.p.) protects against CCl_4_-induced acute liver damage via the inhibition of lipid peroxidation subsequently reduced to free-radical production [220]. The treatment of aloe emodin also ameliorated the inflammatory lesions in liver cells and ultimately reduced the leakage of liver markers L-aspartate-2-oxoglutate-aminotransferase in serum via the inhibition of proinflammatory cytokines TNF-*α* mRNA expression [220]. Later, Woo et al. demonstrated that aloe emodin can also inhibit the activation and proliferation of hepatic stellate in vitro by the reduction of DNA synthesis and inhibition of type I collagen production and sm-*α* (smooth muscle *α*-actin) expression (0.004–0.04 *µ*M/mL), a key liver cell that has an essential role in the pathogenesis of liver fibrosis [221].

Aloin (**53**) is another anthraquinone glycoside that has been reported to isolate from different *Aloe* species including *A. vera* (Figure 15) [222]. Aloin protects against chronic alcoholic liver injury at a dose of 24–72 *µ*M/kg by attenuating lipid accumulation, oxidative stress, and LPS-induced inflammatory response as well as significant reduction of hepatic mRNA expression of CYP2E1 [223]. The molecular mechanism of the reduction of lipid accumulation was observed by the activation of AMP-activated protein kinase-*α*2 (AMPK-*α*2) and downregulation of sterol regulatory element-binding protein-1c (SREBP-1c) expression that has a role in the balance between lipid synthesis and fatty acid oxidation/lipolysis. A recent interesting study conducted by Jung et al. reported the protective effect of aloin against retinal injury associated with liver failure by normalization of Kir4.1 and aquaporin-4 channels in TAA-induced hepatic retinopathy [224].

Embelin (**54**), chemically known as 2,5-dihydroxy-3-undecyl-1,4-benzoquinone, is a natural para-benzoquinone derivative derived from the BHP of *Embelia ribes* that possesses a wide range of medicinal activities including hepaprotective activity (Figure 15) [225]. Sreepriya and Bali investigated the protective effect of embolin (170 *µ*M/kg) against hepatocarcinogenesis induced by N-nitrosodiethylamine- (DENA-) initiated and phenobarbital- (PB-) promoted hepatocarcinogenesis in the rat model. The results showed that embolin has the ability to prevent leakage of hepatic biomarkers, inhibit lipid peroxidation, upregulate antioxidant defense, and reduce the percentage of hepatic hyper plastic nodule incidence and hypoproteinemia in DENA-/PB-treated hepatocarcinogenesis rats [226, 227]. The antioxidant activity of embelin was further confirmed to involve in the protection of liver toxicity in rats [228]. The molecular mechanism of hepatoprotective activity of embelin was not clear; however, a previous study showed that embelin has the ability to modulate Nrf-2/HO-1, MAPK/NF-*κ*B, p53, and STAT3 signaling pathways to regulate cellular oxidative stress, inflammatory response, and apoptosis that might be responsible for its protective effect against hepatotoxin-induced liver damage [229–231].

#### 3.3.5. Fatty Acids

Natural fatty acids are common phytoconstituents in various functional foods that possess different bioactivities and have been used as a supplement to treat different diseases. *trans*-Tetracos-15-enoic acid (TCA) (**55**), a monounsaturated fatty acid, was derived from bioactivity-guided isolation of the dried aerial parts of *Indigofera tinctorial* Linn. that possess hepatoprotective activity in CCl_4_- and APAP-induced liver toxicity in the rat and mice model (Figure 15) [232]. The study demonstrated that TCA has both preventive and curative potential (34–273 *µ*M/kg, p.o) as a hepatoprotective agent and it was comparable to that of the known protective agent silymarin. Pre- and posttreatment of TCA showed significant dose-dependent (12.5–100 mg/kg, p.o) restoration of elevated serum level of liver marker enzymes and inhibited lipid peroxidation as well as upregulated antioxidant enzyme GSH.

#### 3.3.6. Carotenoids

A carotenoid derivative apocarotenoid, known as bixin (**56**), has been isolated from *B. orellana* L. seeds that possess various pharmacological properties (Figure 15) [233]. Pinzón-García et al. reported that bixin (127 *µ*M/kg) and bixin: *β*-cyclodextrin combination could ameliorate nonalcoholic fatty liver steatosis and its associated obesity, hyperglycemia, and hyperlipidemic condition in the high-fat-diet C57BL/6 mice model [234]. The molecular mechanism of its hepatoprotective activity was not clear, but the study demonstrated the hepatoprotective effect of bixin involved the improvement of lipid profile and inhibition of fat accumulation in the liver.

#### 3.3.7. Polysaccharide

Polysaccharide (**57**) is another main bioactive constituent in *A. vera* gel that has been reported to possess a number of biological activities including liver protective activity [235]. Cui et al. demonstrated the supplementation of polysaccharide extracted from *A. vera* against alcohol liver disease (ALD) in a chronic alcohol-feeding mouse model [236]. The hepatoprotective effect of polysaccharide involved with its antioxidant activity increased lipolysis and anti-inflammatory response. The molecular mechanism of lipolysis by polysaccharide was observed by significant upregulation of hepatic expression of lipolytic genes AMPK-*α*2 and PPAR*α*; on the other hand, alcohol-induced inflammation was protected through downregulation of TLR-4 and MyD88 and upregulation of I*κ*B-*α* (nuclear factor of kappa light polypeptide gene enhancer in B-cell inhibitor, alpha) [236].

## 4. Challenges with Bangladeshi Hepatoprotective Plants

Based on ethnomedicinal evidence and practice, plant extracts and their active constituents have the potential to treat liver diseases. This review summarized Bangladeshi plants that have been traditionally used for the treatment of liver diseases, namely, jaundice, ascites, liver cirrhosis, hepatitis, liver enlargement, inflammatory liver, sclerosis of the liver, and other ailments. Literature survey revealed that a number of these plants have been reported to ameliorate liver toxicity or injury induced by various chemicals, drugs, and/or foods, in both in vitro and in vivo settings. Generally, people from rural areas are largely dependent on traditional herbal medicine for their primary healthcare needs, including treatment of liver problem, because of traditional evidence of their effectiveness and safety, as well as lack of access to modern drugs. However, the major challenges of these ethnomedicinal herbal treatments are lack of standardization, quality, efficacy, and taxonomic documentation and toxic effects. A number of BHPs underwent pharmacological and phytochemical analysis in terms of their hepatoprotective activity, although a major portion of these plants is still either underexplored or unexplored. Therefore, there is an urgent need for preclinical and clinical studies of these plants to study their efficacy in the treatment of liver diseases. The resolution for these challenges needs rigorous chemical and clinical research to confirm the potentials of these BHPs and identify their active constituents in the treatment of different liver diseases. Since large percentages of people with liver diseases use botanicals as prophylactics all over the world, a substantial effort is being made in recent years to develop plant-based therapeutics with a novel mechanism of action.

Finally, Bangladesh is located at the juncture of the Indo-Malayan and Indo-China subregion of tropical South-East Asia. With this unique geographical location, the land of Bangladesh (Figure 3) is very fertile for plant growth. About 2.5 million hectares of land is covered by forest which is approximately 17.5% of the total area. Since Bangladesh is in the forefront of global climate change and very susceptible to natural calamities, pollution, and man-made deforestation, these valuable plant resources are already under threat. It is important for governmental and nongovernmental organizations to come forward and preserve these precious plant resources so that proper scientific evaluation and documentation can be carried out before they perish.

## 5. Conclusions

This review summarized 88 Bangladeshi ethnomedicinal plants that have been traditionally used in the treatment of different liver problems, and among these, 64 species have been reported to have hepatoprotective activity either in vivo or in vitro, and 17 species underwent further phytochemical analysis to identify active constituents. Literature review revealed that *A. vera*, *A. paniculata*, *C. fistula*, *C. longa,* and *B. diffusa* with their active compounds, namely, andrographolide (**16**), aloe emodin (**52**), curcumin (**46**), karaviloside III (**41**), catechin (**4**), chebulic acid (**14**), and gallic acid (**12**) were the most promising lead molecules, which have the potential for further development for hepatoprotective drug discovery. The hepatoprotective activity of these plants was reported to act through different mechanisms, including enhancing regeneration of hepatocytes or decreasing degradation/necrosis of liver cells and subsequently reducing leakage or restoring elevated level of serum liver biomarkers, as well as inhibiting lipid peroxidation and upregulating hepatic antioxidant enzyme activities. Induction of apoptosis of injured hepatocytes and protection from cytochrome enzymes were also reported by the liver-protective agents. The molecular mechanism of activity of constituents varied from molecule to molecule, but the activation of Nrf2/HO-1 and inhibition of p38 MAPKs and TLR4/MyD88/NF-*κ*B were the most common pathways revealed from literature survey. Although a number of plants with similar phytoconstituents have also been explored, a good number of BHPs are still unexplored in terms of isolation of active principle(s), as well as scientific validation of their traditional claim as a hepatoprotective agent. Finally, Bangladeshi plants represent a valuable resource for the development of therapeutics; therefore, well-designed and controlled clinical trials need to be executed on traditionally used BHPs, together with the chemical profiling of actives or markers which will establish the efficacy and safety of botanical medicine for liver diseases.

## Figures and Tables

**Table 1:** Traditional use and other information of Bangladeshi medicinal plants used to treat different liver diseases [26, 28].

Name of plants	Local name	Habit	Family	Used parts	Form of use	Diseases
*Allamanda cathartica* L.	Malatilata	Shrub	Apocynaceae	Bark	NA	Ascites
*Alocasia indica* Roxb.	Mankachu	Herb	Araceae	Tuber	NA	Jaundice
*Aloe barbadensis/A. vera* L.	Ghritakumari	Herb	Liliaceae	Leaves	Juice	Jaundice
*Aloe indica* Linn.	Ghritakumari	Herb	Liliaceae	Leaves	Juice	Jaundice
*Alpinia calcarata* Rose.	Bara kulanjan	Herb	Zingiberaceae	Rhizome	NA	Liver disease
*Alpinia nigra* Burtt.	Jangli ada	Herb	Zingiberaceae	Rhizome	Crushed rhizome	Liver disease
*Amaranthus spinosus* Linn.	Katanotey	Herb	Amaranthaceae	Leaves and roots	Decoction	Jaundice
*Anagallis arvensis* Linn.	Blue pimpernel	Herb	Primulaceae	Whole plant	NA	Liver disease
*Andrographis paniculata* Burm. f.	Kalomegh	Herb	Acanthaceae	Leaves	Juice	Liver disease
*Apanamixis polystachya* Wall.	Roina and tiktaraj	Tree	Meliaceae	Bark	NA	Liver disease
*Argenome Mexicana* Linn.	Shialkata	Herb	Papaveraceae	Whole plant	Latex and extract of plants	Jaundice
*Asparagus racemosus* Wild.	Shatamuli	Herb	Liliaceae	Whole plant	NA	Jaundice
*Averrhoa carambola* Linn.	Kamranga	Tree	Oxalidaceae	Fruits	Fresh fruit	Jaundice
*Baliospermum montanum* Wild.	Dantimul	Shrub	Euphorbiaceae	Root	Decoction	Jaundice
*Bixa Orellana* Linn.	Latkan and annato	Tree	Bixaceae	Root	Aqueous extract	Jaundice
*Boerhaavia diffusa* Linn.	Punarnava	Herb	Nyctaginaceae	Leaves and roots	Juice	Jaundice and ascites
*Borassus flabellifer* Linn.	Tal gachh	Tree	Palmae	Fruit	Palm sugar and candy	Liver disease
*Caesalpini*a *bonducella* Linn.	Nata karanja	Shrub	Caesalpiniaceae	Leaves	NA	Liver disease
*Caesalpinia pulcherrima* Linn.	Krishnachura	Tree	Caesalpiniaceae	Leaves	NA	Liver disease
*Cajanus cajan* Linn.	Arhar and tur	Shrub	Papilionaceae	Leaves	Juice	Jaundice
*Calycopteris floribunda* Lam.	Goache lata	Shrub	Combretaceae	Fruits	Juice	Jaundice
*Carthamus tinctorius* Linn.	Kajirah	Herb	Compositae	Flowers	Hot infusion	Jaundice
*Cassia fistula* Linn.	Bandar lathi	Tree	Caesalpiniaceae	Seeds	NA	Jaundice
*Carica papaya* Linn.	Pepe	Tree	Carieaceae	Fruits	Fresh fruits	Liver disease
*Chenopodium album* Linn.	Bethusag	Herb	Chenopodiaceae	Leaves	Juice	Liver disease
*Citrullus colocynthis* Linn.	Makal	Herb	Cucurbitaceae	Roots	NA	Ascites and jaundice
*Citrullus lanatus* Thunb.	Tarmuj	Herb	Cucurbitaceae	Fruits	Fresh fruits	Liver disease
*Clitoria ternatea* Linn.	Aparajita and nila	Herb	Papilionaceae	Seeds	Roasted and powdered seeds	Ascites
*Corchorus olitorius* Linn.	Tosha pat	Herb	Tiliaceae	Leaves	Juice	Ascites
*Corchorus capsularis* Linn.	Deshi pat	Herb	Tiliaceae	Leaves	Juice	Liver disease
*Croton caudatus* Geisel.	Nan-bhantur	Shrub	Euphorbiaceae	Leaves and buds	NA	Liver disease
*Croton oblongifolius* Roxb.	Baragachi	Tree	Euphorbiaceae	Bark and roots	NA	Liver disease
*Curculigo orchioides* Gaertn.	Talamuli	Herb	Amaryllidaceae	Rhizomes	NA	Jaundice
*Curcuma longa* Linn.	Halud and haldi	Herb	Zingiberaceae	Rhizome	Essential oils	Liver disease
*Cucumis melo* Linn.	Kharmuj	Herb	Cucurbitaceae	Seeds	Extract of seeds	Ascites
*Cuscuta reflexa* Roxb.	Swarnalata	Herb	Convolvulaceae	Whole plant	Crushed of plant	Jaundice
*Daucas carota* Linn.	Gajor	Herb	Umbelliferae	Rhizome	Fresh rhizome	Jaundice
*Ecbolium viride* Forsk.	Nilkanta	Shrub	Acanthaceae	Roots	NA	Jaundice
*Eclipta alba* Linn.	Kesuti and keshraj	Herb	Compositae	Whole plant	Decoction/juice	Jaundice
*Elephantopus scaber* Linn.	Gojilata	Herb	Compositae	Roots	Crushed roots	Liver disease
*Eleusine indica* Linn.	Malangakuri	Herb	Gramineae	Roots	NA	Liver disease
*Embelia ribes* Bur.f.	Biranga	Shrub	Myrsinaceae	Fruits	NA	Jaundice
*Euphorbia tirucalli* Linn.	Lanka sij	Shrub	Euphorbiaceae	Whole plant	Juice	Jaundice
*Flacourtia jangomas* Lour.	Paniala	Shrub	Flacourtiaceae	Fruit	NA	Liver disease
*Glycosmis pentaphylla* Corr.	Motkilagachh	Shrub	Rutaceae	Leaves	Infusion	Jaundice
				Whole plant	Methanolic extract	Liver disease
*Hedyotis corymbosa* Linn.	Khetpapra	Herb	Rubiaceae	Enter plant	NA	Jaundice
*Hypericum japonicum* Thunb.	Bassanta	Herb	Hypericaceae	Whole plant	NA	Liver disease
*Hygrophila auriculata* Schum.	Talmakhna	Herb	Acanthaceae	Seeds	Methanolic extract	Liver disease
*Indigofera tinctora* Linn.	Neel and indigo	Shrub	Papilionaceae	Roots	NA	Hepatitis
*Ipomoea aquatica* Forsk.	Kalmishak	Herb	Convolvulaceae	Stems/leaves	Fresh juice or cooking	Liver complaints
*Justicia gendarussa* Burm.	Jagatmadan	Shrub	Acanthaceae	Roots	Decoction	Jaundice
*Kalanchoe pinnata* Lam.	Patharkuchi	Herb	Crassulaceae	Leaf	Juice	Jaundice
*Lagenaria siceraria* Mol.	Lau and kodu	Shrub	Cucurbitaceae	Leaves	Decoction with sugar	Jaundice
*Lawsonia inermis* Linn.	Mehedi	Tree	Lythraceae	Bark	Decoction	Jaundice
*Mentha arvensis* Linn.	Pudina	Herb	Labiatae	Aerial part	Juice	Jaundice
*Meyna spinosa* Roxb.	Moyna	Shrub	Rubiaceae	Fruits	Decoction of frozen fruits	Liver disease
*Momordica charantea* Linn.	Uchahe and karalla	Herb	Cucurbitaceae	Leaves/fruits	Juice	Jaundice
*Moringa oleifera* Lamk.	Sajnagachh	Tree	Moringaceae	Fruits	NA	Diseases of the liver
*Mussaenda glabrata* Hutch.	Nagabali	Shrub	Rubiaceae	Leaves	Crushed with milk	Jaundice
*Nelumbo nucifera* Gaertn.	Podma and lotus	Herb	Nymphaeaceae	Flowers	Crushed of flower	Liver disease
*Nymphoides cristatum* Lour.	Chandmala	Herb	Gentianaceae	Whole plant	NA	Jaundice
*Ocimum basilicum* Linn.	Babuitulshi	Shrub	Labiatae	Leaves/flowers	Juice	Sclerosis of the liver
*Paederia foetida* Linn.	Gondhabadali	Shrub	Rubiaceae	Roots and barks	NA	Liver pain
*Pavetta indica* Linn.	Kukurchura	Shrub	Rubiaceae	Root	Pulverized with ginger and rice water	Ascites
*Phyllanthus acidus* Linn.	Horbori and orbori	Tree	Euphorbiaceae	Fruits	Fresh fruits	Tonic to the liver
*Phyllanthus emblica* Linn.	Amlaki	Tree	Euphorbiaceae	Fruits	Fresh fruits	Jaundice
*Phyllanthus freternus* Web.	Bhui-amla	Herb	Euphorbiaceae	Roots	Fresh roots	Jaundice
*Piper longum* Linn.	Pipul	Shrub	Piperaceae	Fruits	Unripe fruits	Jaundice
*Piper nigrum* Linn.	Golmorich	Shrub	Piperaceae	Fruits	Crushed of fruits	Ascites
*Plumbago indica* Linn.	Lalchita	Herb	Plumbaginaceae	Root	NA	Liver disease
*Portulaca oleracea* Linn.	Nuneshak	Herb	Portulacaceae	Whole plant	Juice	Liver disease
*Rumex vesicarius* Linn.	Tok-palong	Herb	Polygonaceae	Seeds	Fresh seeds	Jaundice
*Saccharum officinarum* Linn.	Aakh and kuishar	Shrub	Gramineae	Stem	Juice	Jaundice
*Semecarpus anacardium* Linn. f.	Bhela	Tree	Anacardiaceae	Ripe fruits	Fresh ripe fruits	Ascites
*Solanum nigram* Linn.	Phuti begoon	Herb	Solanaceae	Aerial parts	Juice	Liver enlargement
*Solanum torvum* Sw.	Tit begoon	Shrub	Solanaceae	Leaves/fruits	Extract of fruits and leaves	Liver enlargement
*Sonchus wightianus* DC.	Bon palong	Herb	Compositae	Roots	NA	Jaundice
*Spinacia oleracea* Linn.	Palong shak	Herb	Chenopodiaceae	Seeds	NA	Liver inflammation
*Syzygium jambos* Linn.	Golap jam	Tree	Myrtaceae	Fruits	Fresh fruits	Liver complaints
*Tamarix troupii* Hole.	Bon jhau	Shrub	Tamaricaceae	Leaves	Ash of leaves	Hepatoprotective
*Tephrosia purpurea* Linn.	Sarpunkha	Herb	Papilionaceae	Leaves	NA	Jaundice
*Terminalia arjuna* Roxb.	Arjun gach	Tree	Combretaceae	Bark	Powdered bark	Liver cirrhosis
*Terminilia bellirica* Roxb.	Bohera	Tree	Combretaceae	Fruits	Decoction	Hepatitis
*Terminilia chebula* Retz.	Hartaki	Tree	Combretaceae	Fruits	Decoction	Jaundice
*Tirospora cordifolia* Linn.	Guloncha lata	Shrub	Menispermaceae	Leaves/stems	NA	Jaundice
*Trianthema portulacastrum* Linn.	Swet punarnava	Herb	Aizoaceaee	Roots	Decoction	Liver troubles
*Trichosanthes dioica* Roxb.	Potol	Herb	Cucurbitaceae	Leaves	Juice	Liver enlargement
*Wedelia chinensis* Merr.	Kesharaj	Herb	Compositae	Whole plant	NA	Liver enlargement

**Table 2:** Hepatoprotective activity of Bangladeshi medicinal plants.

Plant name	Extract and plant part used	Test model	Dose	Route	Hepatoameliorative effects	Ref.
*A. barbadensis*	AQ extract of leaves	APAP-induced hepatotoxicity in albino rats	250 and 500 mg/kg	Oral	↓ the elevated AST, ALT, and ALP levels and restored the depleted liver thiol levels	[110]
	AQ extract of gel from leaves	Alcohol-induced liver toxicity in rat	1 mL/kg	Oral	↓ the elevated levels of aminotransaminases, ALP, and TB and maintained normal hepatocyte architecture integrity	[111]
	Fresh leaves extract	Lindane- (LD-) induced hepatotoxicity in rat	1 mL/kg	Oral	↓ the elevated levels of SGPT, SGOT, *γ*-GT, and ALP	[112]
	Fresh AQ leaves extract	Isoniazid- (INZ-) and rifampicin- (RMP-) induced liver toxicity in rats	50 mg/kg	Oral	↓ the elevated AST, ALT, ALP, acid phosphatase (ACP), TB, total protein (TP), total albumin (TA), and total globulin (TGb)	[113]
	Fresh AQ leaves extract	CCl_4_-induced hepatic injury in rat	60 mg/kg	Oral	↓ the elevated AST, ALT, *γ*-GT, and ↑ the liver antioxidant enzyme GSH	[114]
	ACTIValoe®N-931 (mixture of *A. vera* and *Silybum marianum*)	CCl4-induced acute hepatotoxicity in rats	85, 170, and 340 mg/kg	IP	↓ the elevated aminotransferase levels and lipid peroxidation and ↑ the liver enzyme GSH, as well as ↓ the tumor necrosis factor-*α* (TNF-*α*), inducible nitric oxide synthase (iNOS), cyclooxygenase-2 (COX-2), and mRNA expressions	[115]
	Fresh juice	Acute hepatitis in a 16–65 yrs of age human subject (a clinical diagnosis model)	20 mL juice twice daily for 6 weeks	Oral	↓ the elevated bilirubin, ALT, and AST	[116]

*A.* indica	Hydroalcoholic extract of leaves	CCl_4_- and APAP-induced hepatotoxicity in rats	250 and 500 mg/kg	Oral	↓ the elevated liver marker enzymes, cholesterol, serum protein, and albumin as well as maintained normal hepatocyte architecture integrity	[237]
	EtOH and AQ extract of tuber	CCl_4_-induced hepatic injury in rat	200 mg/kg	Oral	↑ the liver enzyme GSH, SOD, and CAT as well as ↓ the elevated ALT, AST, and MDA	[238]

*A. nigra*	MeOH extract of leaves	CCl_4_-induced hepatic injury in rat	300 mg/kg	Oral	↓ the elevated SGOT, SGPT, ALP, TB, and TP	[239]

*A. spinosus*	50% EtOH extract of the whole plant	CCl_4_-induced hepatic damage in rat	100, 200, and 400 mg/kg	Oral	Reduced oxidative stress via induction of antioxidant enzymes SOD, CAT, and GSH and inhibited MDA, as well as restored the elevated level of serum AST, ALT, ALP, and TB	[125]
	MeOH extract of seeds	Deltamethrin- (DLM-) induced liver injury in rats	15 mg/kg	Oral	↑ the liver enzyme SOD, CAT, GSH, and GPx and ↓ the elevated MDA ALT, ALP, and LDH	[240]
	50% EtOH extract of the whole plant	GNH2-/lipopolysaccharide-induced rat liver injury	400 mg/kg	Oral	↓ the elevated ALT, AST, ALP and *γ*-GTP, and serum bilirubin (SB)	[127]
	MeOH extract of the whole plant	APAP-induced hepatotoxicity in albino rats	200 mg/kg	Oral	↓ the elevated SGOT, SGPT, ALP, TB and lipid peroxidation as well as ↑ the liver enzyme CAT and GSH	[126]

*A. paniculate*	EtOH extract of leaves	Thioacetamide- (TAA-) induced liver cirrhosis in rats	250 and 500 mg/kg	Oral	↓ the elevated ALT, AST, and ALP and normalized cellular ROS level and proliferation	[77]
	EtOH extract of leaves	CCl_4_-induced hepatic injury in rat	300 mg/kg	Oral	↓ the elevated SGPT and ALP, liver weight, and volume	[78]
	AQ extract of leaves	Hexachlorocyclohexane- (BHC-) induced hepatotoxicity in mice	12 mg/kg	Oral	↓ the elevated ALT, AST, ALP and↓ the activity of *γ*-glutamyl trans peptidase (*γ*-GTP), and lipid peroxidase (LPO)	[79]
	EtOH extract of leaves	APAP-induced hepatotoxicity in mice	100–200 mg/kg	Oral	↓ the elevated GPT, GOT, ALP, TB, and LPO, as well as ↑ the liver enzyme SOD, CAT, GSH, and GPx	[80]

*A. polystachya*	EtOH leaf extracts	CCl_4_-induced hepatic injury in rat	50 mg/kg	Oral	↓ the elevated AST, ALT, ALP, ACP, and LDH	[241]

*A. mexicana*	AQ extract of leaves	CCl_4_-induced hepatitis in rats	250 mg/kg	Oral	↓ the elevated SGOT, SGPT, ALP, and direct bilirubin	[128]
	Leaf powder suspension	CCl_4_-induced hepatic injury in rat	125, 250, and 500 mg/kg	Oral	↓ the elevated AST, SGOT, ALT, SGPT, ALP, TB, and direct bilirubin	[129]
	MeOH and AQ extract of aerial parts	CCl_4_-induced hepatic injury in rat	100, 200, and 400 mg/kg	Oral	↓ the elevated SGOT, SGP, and ALP	[130]

*A. racemosus*	AQ root extract and its fraction	CCl_4_-induced formation of lipid peroxides in the rat liver	300 mg/kg	Oral	↓ the elevated SGPT, SGOT, ALP, TB, and MDA, as well as ↑ the liver enzyme SOD, CAT, and GSH	[242]
	Plant powder	APAP-induced hepatotoxicity in rats	500 mg/kg	Oral	↓ the elevated AST, ALT, ALP, and ↑ the liver enzyme SOD and CAT	[243]
	NA	INZ-induced liver toxicity in rats	50 mg/kg	Oral	↓ the elevated ROS via inhibition of hepatic CYP2E1 activity and ↑ the liver enzyme GSH	[244]

*A. carambola*	AQ fruit extract	CCl4-induced hepatic injury in mice	0.9 g/kg	Oral	↓ the elevated ALT, AST, ALP, and ↑ the liver enzyme GSH	[245]
	EtOH extract of fruits	Chemically induced hepatocellular carcinoma in mice	05, 15, 25, 50, and 75 mg/kg	Oral	↓ the tumor incidence, tumor yield, tumor burden, and LPO, as well as ↑ the liver enzyme SOD and CAT	[246]
	MeOH extracts of ripe fruits	APAP- and D-galactosamine-induced hepatotoxicity in rats	100 mg/kg	Oral	↓ the elevated SGOT, SGPT, and TB	[94]

*B. montanum*	MeOH extract of root	TAA-induced liver toxicity in rats	100, 200, and 300 mg/kg	Oral	↓ the elevated GOT, SGPT, ALP, TB, TG, TP, and albumin	[34]
	EtOH and AQ root extract	APAP-induced liver toxicity in rats	100–2000 mg/kg	Oral	↓ the elevated oxidative stress and GPT, GOT, and ALP	[35]
	MeOH and ethylmetyl ketone subfraction of root	CCl_4_-induced hepatotoxicity in rats	50, 100, and 150 mg/kg	Oral	↓ the elevated GOT, GPT, and TP	[36]
		In vitro hepatocyte viability	100, 500, and 1000 *μ*g/mL	Cell culture	↑ the viability of hepatocyte	
	MeOH subfraction of root	APAP-induced hepatotoxicity in rats	50, 100, and 150 mg/kg	Oral	↓ the elevated oxidative stress and GPT, GOT, and TP	[37]
		In vitro hepatocyte viability	100, 500, and 1000 *μ*g/mL	Cell culture	↑ the viability of hepatocyte and ↓ the elevated level of cellular TP	

*B. orellana*	MeOH extract of aerial parts	CCl4-induced hepatic injury in rats	500 mg/kg	Oral	↓ the elevated ALT, SGPT, AST, SGOT, and cholesterol	[95]
	Seed oil	CCl4-induced hepatic injury in rats	0, 1, 5, and 10%	Oral	↓ the elevated liver biomarker enzymes, TB, and LPO	[132]
	Petroleum ether (PE), MeOH, and AQ leaves extracts	CCl4-induced hepatic injury in rats	250, 500, 750, and 1000 mg/kg	Oral	↓ the elevated TB, direct bilirubin, ALT, AST, ALP, and TP	[131]
	50% EtOH extract of seeds	Ethanol-induced acute hepatotoxicity in rats	200 and 400 mg/kg	Oral	↓ the elevated AST, ALT, ALP, TB, and LDH	[133]

*B. diffusa*	AQ extract of root	TAA-induced hepatotoxicity in rats	2 mL/kg	Oral	↓ the elevated GOT, GPT, ACP, and ALP, but not LDH and bilirubin	[247]
	EtOH extract of root	Ethanol-induced liver damage in rats	150 mg/kg	Oral	↓ the elevated AST, ALT, ALP, LDH, and *γ*-GTP to the normal level	[248]
	MeOH (85%) extract of root and the aerial part	Ibuprofen- (IB-) induced hepatotoxicity in rats	500 mg/kg	Oral	↓ the elevated ALT, AST, ALP, and TB and ↑ the liver enzyme SOD, CAT, GPx, and GST, as well as normalized liver histological changes	[134]
	EtOH extract of the whole plant	CCl_4_-induced hepatotoxicity in mice	100, 200, and 300 mg/kg	Oral	↓ the elevated ALT, AST, ALP, ACP, LDH, *γ*-GT, and TB and inhibit LOP, as well as normalized liver histological changes	[136]
	EtOH (50%) extract of the whole plant	CCl_4_-induced hepatotoxicity in rats	2000 mg/kg	Oral	Protective activity via protection of drug-metabolizing enzymes and ↓ the elevated SGOP and SGPT as well as increased bile flow to the liver	[135]

*C. cajan*	MeOH extract of aerial parts	CCl_4_-induced liver injury in rats	500 mg/kg	Oral	↓ the elevated ALT, SGPT, AST, SGOT, and cholesterol	[95]
	EtOH extract of leaves	D-galactosamine-induced hepatitis in rats	100 mg/kg	Oral	↑ the liver enzyme CAT, SOD, GPx, and GST and ↓ the elevated AST and ALT	[96]
	MeOH-AQ fraction of leaves	Alcohol-induced rat liver damage	50 mg/kg	Oral	↓ the elevated liver marker enzymes and ↑ the liver enzyme activities. Molecular mechanism involved the upregulation of UDP-glucuronosyl transferase-2B (UGT2B) expression and activation of Nrf2	[97]
	MeOH extract of tender leaves	APAP and D-galactosamine induced hepatic injury in rats	100 mg/kg	Oral	↓ the elevated SGOT, SGPT, and TB	[94]

*C. floribunda*	CHCl_3_ fraction of MeOH extract of stem	CCl4-induced hepatotoxicity in rats	100 and 200 mg/kg	Oral	↓ the elevated SGOT, SGPT, ALP, and TB, as well as cellular protection of centribular necrosis and vacuolization	[249]

*C. tinctorius*	Seed oil	Alloxan-induced liver toxicity in type 1 diabetic rats	200 mg/kg	Oral	↓ the elevated blood glucose, TC, TGs, LDL, ALT, AST, and ALP and increased the level of HDL	[61]
	MeOH extract of flowers	CCl4-induced liver injury in rats	200 mg/kg	Oral	↓ the elevated ALT, ALP, AST, MDA, TB, and inflammatory cytokines (TNF-*α* and IL-6), as well as ↑ the liver enzyme SOD, CAT, and GSH	[60]
	MeOH extract of leaves	CCl_4_-induced liver toxicity in rats	150 and 300 mg/kg	Oral	↓ the elevated blood ALT, AST, and ALP	[62]

*C. fistula*	EtOH leaf extract	Diethyl nitrosamine- (DEN-) induced liver toxicity in rats	500 mg/kg	Oral	↑ the liver enzyme SOD and CAT and ↑ the liver enzyme LPO, AST, ALT, ALP, LDH, *γ* –GT, and TB	[103]
	n-Heptane extract of leaves	APAP-induced hepatotoxicity in rats	400 mg/kg	Oral	↓ the elevated SGOT, SGPT, TB and ALP	[98]
	n-Heptane extract of leaves	CCl4 with liquid paraffin (1 : 1)-induced liver injury in rats	400 mg/kg	Oral	↓ the elevated SGOT, SGPT, TB, and ALP	[99]
	Hydroalcoholic extract of fruit	Bromobenzene-induced liver toxicity in mice	200, 400, 600, and 800 mg/kg	Oral	Dose-dependently ↓ the elevated AST, ALT, ALP, and TB	[101]
	AQ extract of fruit pulp	CCl4-induced liver injury in rats	200 mg/kg	Oral	↓ the elevated AST, ALT, ALP, and TB and increase in TP	[102]
	MeOH extract of seeds	APAP-induced hepatitis in rats	200 and 400 mg/kg	Oral	↓ the elevated SGOT, SGPT, ALP, and TB to the normal levels	[100]
	EtOH extract of leaves	INZ- and RIF-induced liver toxicity in rats	400 and 500 mg/kg	Oral	↓ the elevated oxidative stress and ALT, AST, ALP, and TB	[104]

*C. bonducella*	AQ extract of leaves	CCl_4_-induced chronic rat hepatotoxicity	NA	Oral	↓ the elevated ALT, AST, ALP, TB, and prothrombin time (PT)	[107]
	EtOH extract of leaves	CCl_4_-induced hepatotoxicity in rat	250 and 500 mg/kg	Oral	↓ the elevated AST, ALT, ALP, TB, and MDA with an ↑ the liver enzyme CAT and GPx	[105]
	MeOH leaf extract	Gentamicin-induced rat liver toxicity	250 and 500 mg/kg	Oral	↑ the liver enzyme ALT, AST, ALP, TB, and TP	[106]
	MeOH extract of leaves	CCl_4_-induced chronic rat hepatotoxicity	50, 100, and 200 mg/kg	Oral	↑ the liver enzyme SGPT, SGOT, ALP, TB, uric acid, and LPO whereas reduced oxidative stress via ↑ the liver enzyme SOD, CAT, GSH, vit. C, vit.E, and protein	[109]
	MeOH extract of leaves	APAP-induced liver damage in rats	50, 100, and 200 mg/kg	Oral	↓ the elevated liver marker enzymes, bilirubin, and LPO, as well as ↑ the liver enzyme GSH, SOD, CAT, and protein	[108]

*C. papaya*	MeOH extract of stalk	CCl4-induced liver damage in rats	20, 40, 60, 80, and 100 mg/kg	Oral	↓ the elevated TP, AST, and ALT	[138]
	AQ extract of ripe seed	CCl4-induced hepatotoxicity in rats	100, 200, and 300 mg/kg	Oral	↓ the elevated ALT, AST, ALP, and bilirubin	[139]
	AQ extract of leaf and unripe fruits	CCl4- and APAP-induced hepatotoxicity in rats	100 and 300 mg/kg	Oral	↑ the liver enzyme GSH, SOD, and CAT, as well as ↓ the elevated AST, ALT, ALP, LDH, and MDA	[137]
	AQ seed extract of unripe fruit	CCl4-induced hepatotoxicity in rats	100–400 mg/kg	Oral	↓ the elevated liver marker enzyme (ALT and AST), serum lipids (TG, TC, HDL-c, LDL-c, and VLDL-c), and serum proteins (TP and ALB)	[141]
	AQ and EtOH extracts of dried fruit	CCl4-induced hepatotoxicity in rats	250 mg/kg	Oral	↓ the elevated ALT, AST, and ALP	[140]

*C. album*	EtOH extract of leaves	EtOH-induced hepatotoxicity in rat	100, 200, and 400 mg/kg	Oral	↓ the elevated liver marker enzymes and LPO, as well as ↑ the liver enzyme GSH, SOD, and CAT	[250]
	AQ extract of leaves	CCl4-induced liver fibrosis in rats	100, 200, and 400 mg/kg	Oral	↓ the elevated SGPT, SGOT, ALP, LDH, TC, and TB	[251]
	EtOH extract of leaves	EtOH-induced hepatotoxicity in rat	200, 400, and 600 mg/kg	Oral	↓ the elevated AST, SGOT, ALT or SGPT, ALP, TB, TC, and LPO and ↑ the liver enzyme SOD, CAT, and GSH	[252]
	Alcoholic and AQ extracts of the aerial	APAP-induced hepatotoxicity in rats	200 and 400 mg/kg	Oral	↓ the elevated transaminases, alkaline phosphatase, and bilirubin content	[253]

*C. colocynthis*	MeOH extract of fruits	APAP-induced hepatotoxicity in rat	300 mg/kg	Oral	↓ oxidative stress via the antioxidant mechanism and ↓ the elevated TB, SGOT, SGPT, and ALP	[40]
	EtOH fruit extract	Cisplatin-induced hepatorenal toxicity in rats	100, 200, and 400 mg/kg	Oral	↓ the elevated MDA and nitrite levels, as well as ↑ the liver enzyme GSH, CAT, and SOD	[42]
	Hydroalcoholic fruit extract and its subfraction	CCl_4_-induced and lipopolysaccharide-induced hepatotoxicity in mice	400 mg/kg	Oral	↓ the elevated AST and ALT	[43]
	EtOH extract of roots	CCl_4_-induced hepatic toxicity in rats	100 mg/kg	Oral	↓ the elevated GPT, GOT, ALP, and bilirubin	[45]
	EtOH fruit extract	Polluted water-induced hepatic damage in rats	100 and 200 mg/kg	Oral	↓ the elevated AST, ALT, ALP, TP, and bilirubin	[44]
	EtOH fruit extract	APAP-induced hepatic injury in rats	50, 100, and 200 mg/kg	Oral	↑ cell membrane stabilization, hepatic cell regeneration and ↓ the elevated AST, ALT, and ALP	[41]

*C. lanatus*	Aqueous EtOH seed extract	CCl_4_-induced hepatic fibrosis in mice	100, 200, 400, and 800 mg/kg	Oral	Improving drug metabolizing enzyme activity, ↓ the elevated AST, ALT, HA, and LN, as well as ↑ the liver enzyme SOD and GP_x_. Molecular mechanism involved the inhibition of *α*-SMA and TGF-*β*1 protein expression	[47]
	Juice of pulp	Ethanol-induced hepatic oxidative stress in rats	4 mL/kg	Oral	↓ the elevated MDA and ↑ the liver enzyme CAT	[46]
	EtOH leaf extract	CCl4-induced hepatic toxicity in rats	500, 1,000, and 1,500 mg/kg	Oral	↓ congestion and necrosis, as well as ↓ the elevated AST, ALT, ALP, and bilirubin	[50]
	MeOH seed extract	APAP-induced liver injury in rats	200 and 400 mg/kg	Oral	↓ the elevated AST, ALT, ALP, and bilirubin	[48]
	AQ and MeOH extracts of seeds	Salt- and APAP-induced hepatic toxicity in female rats	200 and 400 mg/kg	Oral	Exerts antioxidant effects and healing with rejuvenating effects on the liver, as well as protects hepatocytes	[49]

*C. orchioides*	MeOH extract of rhizome	CCl4-induced hepatopathy in rats	70 mg/kg	Oral	↑ the liver enzyme SOD, CAT, GPx, and GR	[254]

*C. longa*	Crude extract of rhizome	INZ-RIF-pyzinamide (PZA)-induced hepatic injury in Guinea pigs	200 mg/kg	Oral	↓ the elevated AST, ALT, and normalized liver histological changes	[120]
	MeOH extract of rhizome	D-galactosamine- (GNH2-) induced liver injury in mice	100 mg/kg	Oral	↑ the liver enzyme SOD, glutathione-s-transferase (GST), CAT and SOD, and GSH with a ↓ in the elevated LPO, AST, ALT, and ALP	[121]
	MeOH extract of rhizome	Alloxan-induced liver injury in rabbits	2000 mg/kg	Oral	Improves the levels of serum glucose, serum transaminases and antioxidant activity	[124]
	EtOH extract of rhizome	TAA-induced liver cirrhosis in rats	250 and 500 mg/kg	Oral	↓ the elevated MDA, AST, ALT, ALP, nitrotyrosine, and urinary 8-hydroxyguanosine and ↑ the liver enzyme SOD and CAT, as well as restored the elevated cytokines TGF-*β*1, TNF-*α*, and induction of apoptosis	[119]
	EtOH extract of rhizome	Mercuric chloride-induced hepatotoxicity and oxidative stress in rat	200 mg/kg	Oral	↓ the elevated transaminase, ALP, LDH, TB, *γ*-GT, and TG, as well as a protective effect on drug metabolizing CYP 2E1 enzymes, viz., aniline hydroxylase (AH) and amidopyrine-N-demethylase (AND)	[123]
	50% EtOH extract of rhizome	CCl_4_-induced acute hepatic stress in rat	100, 200, and 300 mg/kg	Oral	↓ the elevated ALT, AST and ALP, and LPO	[122]

*C. reflexa*	AQ extracts	CHCl_3_-, EtOH-, and APAP-induced hepatotoxic rat	50, 100, and 200 mg/kg	Oral	↓ the elevated SGOT, SGPT, and ALP restore to the normal level	[255]

*C. melo*	MeOH extract fruit	RIF-INZ induced hepatotoxicity in rat	100, 250, and 500 mg/kg	IP	↓ the elevated ALT, AST, ALP, LPO, TB, and TP, as well as ↑ the liver enzyme GPx, GRD, SOD, CAT, and GSH	[256]

*C. ternatea*	MeOH extract of leaf	APAP-induced liver injury in mice	200 mg/kg	Oral	↓ the elevated ALT, AST, and TB	[257]

*D. carota*	Oil extract and its fraction of tuber root	CCl_4_-induced hepatotoxicity in rats	50, 100, and 200 mg/kg	IP	↑ the liver enzyme SOD, CAT, and GST, as well as ↓ the elevated AST and ALP	[144]
	AQ extract of tuber roots	APAP-, INZ-, and EtOH-induced liver injury in rats	25 mL/kg	Oral	↓ the elevated AST, ALT, TB, and PT	[142]
	AQ extract of tuber roots	LD-induced hepatotoxicity in rats	25 mL/kg	Oral	↓ the elevated AST, ALT, ALP, thiobarbituric acid reactive substances, TC, TG, and LDL‐cholesterol. It also restored the depressed antioxidant.	[143]
	MeOH extract of seeds	TAA-induced liver toxicity in rats	200 and 400 mg/kg	Oral	↑ the liver enzyme SOD, CAT, GPx, GST, and GR and ↓ the elevated LPO, SGPT, SGOT, and ALP	[258]

*E. viride*	EtOH extract of roots	APAP-induced liver injury in rat	200, 400 mg/kg	Oral	↓ the elevated SGPT, SGOT, ALP, TB and TG	[259]
	MeOH extract of whole plant	CCl_4_- and APAP-induced hepatotoxicity in rats	300 mg/kg	Oral	↓ the elevated SGOT, SGPT, ALP, and TB, as well as ↑ the liver enzyme GSH level	[260]

*E. alba*	MeOH leaves and CHCl_3_ root extract	CCl_4_-induced liver damage in rats	250 mg/kg	Oral	Reduced lysosomal enzyme in blood	[66]
	AQ leaf extract	CCl_4_-induced hepatic injury in rats	250 mg/kg	Oral	↓ the elevated AST, ALT, and ALP and ↑ the liver enzyme SOD, CAT, GPx, and GST	[65]
	AQ leaf extract	EtOH-induced oxidative stress on liver in rats	250 mg/kg	Oral	↓ the elevated AST, ALT, and ALP, as well as ↑ the liver enzyme SOD and CAT	[67]
	EtOH extract of aerial parts	CCl4-induced hepatotoxicity in rat and mice	62.5–500 mg/kg	Oral	↑ protection of liver drug metabolizing enzyme, ↑ bromosulphalen (BSP) clearance, and ↓ the elevated AST, ALT, and TB	[64]

*E. scaber*	EtOH extract of leaves	Alcohol-induced liver damage in mice	3, 15, and 30 mg/kg	Oral	↓ the elevated ALT, AST, and ALP level to near the normal value	[68]
	MeOH extract of root	CCl_4_-induced liver damage in rats	75 and 150 mg/kg	Oral	↑ antioxidant mechanisms, especially its free-radical-scavenging activity, as well as ↓ the elevated ALT, AST, and ALP	[70]
	Dry root powder in water	CC1_4_-induced chronic liver dysfunction in rat	250–1500 mg/kg	Oral	↓ the elevated AST, ALT, and ALP	[71]

*E. indica*	AQ extract of aerial parts	CCl_4_-mediated oxidative hepatic damage in rats	150 and 300 mg/kg	Oral	↑ liver enzymes SOD, CAT, GSH, GST, GR, and QR, as well as ↓ the elevated MDA, ALT, and AST	[261]

*E. tirucalli*	AQ bark extract	CCl_4_-induced hepatic damage in rats	125 and 250 mg/kg	Oral	↓ the elevated liver markers, enzymes, bilirubin, cholesterol, triglycerides, and LPO, as well as ↑ liver enzyme GSH	[262]

*G. pentaphylla*	MeOH extract of leaves	APAP-induced hepatic damage in mice	200 and 400 mg/kg	Oral	↓ the elevated ALT, AST, ALP, TP, and liver weight	[263]

*H. corymbosa*	EtOH extract of the whole plant	CCl_4_-induced hepatotoxicity in rats	500–3000 mg/kg	Oral	↓ the elevated SGOT and SGPT	[264]

*H. japonicum*	AQ extract of the whole plant	CCl_4_-induced acute hepatotoxicity in mice	0.5–4.5gm raw material/kg	Oral	↓ the elevated AST, ALT, and TB	[265]

*H. auriculata*	Alkaloidal fraction of MeOH leaves extract	CCl_4_-induced toxicity in rat	80 mg/kg	Oral	↓ the elevated AST, ALT, TG, ALP, TB, and LDH	[266]

*I. tinctora*	AQ extract of leaves	INZ-induced hepatotoxicity in albino rats	5 and 10 mL/kg	Oral	↓ the elevated AST, ALT, ALP, TB, and TP	[267]

*K. pinnata*	EtOH extract of leaves	CCl4-induced hepatotoxicity in rat	100 mg/mL	Oral	↓ the elevated SGOT, SGPT, ALP, and TB, as well as protect liver drug metabolizing enzyme	[268]

*L. inermis*	AQ extract of leaves	CCl4-induced hepatotoxicity in rat	1 mL/kg	Oral	↓ the elevated SGPT, SGOT, ALP, and TB	[269]
	50% EtOH extract of bark	CCl4-induced oxidative stress in rats	250 and 500 mg/kg	Oral	↑ liver antioxidant enzymes and metabolizing enzymes, as well as ↓ the elevated SGPT, SGOT, and LDH	[270]
	Hydroalcoholic extract of barks	CCl4-induced liver toxicity in rats	20 mg/mL	Oral	Upregulation of liver-metabolizing enzymes and restored the elevated level of serum liver biomarkers	[271]
	MeOH extract of leaves	CCl4-induced hepatotoxicity in rat	100 and 200 mg/kg	Oral	↓ the elevated AST, ALT, ALP, and bilirubin	[272]
	Butanolic fraction of leaves	2-Acetylaminofluorene- (2-AAF-) induced hepatic damage in rats	100, 200, and 400 mg/kg	Oral	↓ the elevated SGOT, SGPT, ALP, and LPO as well as restored the normal liver architecture	[273]

*M. arvensis*	CHCl_3_, EtOH, and AQ extract of leaves	CCl_4_-induced liver damage in rats	375 mg/kg	Oral	↓ the elevated SGOT, SGPT, ALP, and TB and also preserved the liver tissue as normal	[274]

*M. charantea*	Hydroalcoholic extract of leaves	CCl_4_-induced hepatopathy in rats	100 and 200 mg/kg	Oral	↓ the elevated SGOT, SGPT, ALP, and TB	[54]
	AQ extract of fruit	Liver injury in restraint-stressed mice	250, 500, and 750 mg/kg	Oral	↓ the elevated liver AST, ALT, and NO, well as ↑ activities of mitrochondrial respiratory chain complex I and II	[56]
	Hydroalcoholic extract of leaves	CCl_4_-induced hepatopathy in rats	100 and 200 mg/kg	Oral	↓ the elevated SGOT, SGPT, ALP, and TB	[55]
	AQ extract of fruit	Cyclophosphamide- (CP-)induced hepatotoxicity in rats	300 mg/kg	Oral	↓ the elevated AST, ALT, ALP, TP, LDH, and TB to normal values	[51]
	AQ extract of leaves	CCl_4_-induced hepatotoxicity in rats	200 and 400 mg/kg	Oral	Restored the elevated level of AST, ALT, ALP, and TB, as well as ↑ liver enzyme SOD and CAT	[53]
	Alcoholic extract of fruit	Ammonium chloride-induced hyperammonemic rats	300 mg/kg	Oral	Restored the hepatic elevated level of AST, ALT, and ALP, as well as ↑ liver antioxidant enzyme SOD, GPx, and CAT	[57]

*M. oleifera*	MeOH extract of leaves	Streptozotocin- (STZ-) induced hepatotoxicity in diabetic rats	250 mg/kg	Oral	↓ the elevated AST, ALT, ALP, TP, and TB to normal values, well as restored the cytokine level including IL-6, monocyte chemoattractant protein-**1** (MCP-1), and TNF-*α*	[275]

*N. nucifera*	50% EtOH extract of flower	CCl_4_- and APAP-induced hepatopathy in rats	200 and 400 mg/kg	Oral	↓ the elevated AST, ALT, ALP, and TB	[276]

*N. cristatum*	50% EtOH extract of the whole plant	CCl4-induced acute hepatic damage in rats	500 mg/kg	Oral	↓ the elevated SGOT, SGPT, ALP, and TB and preserved hepatic tissues to normal	[277]

*O. basilicum*	EtOH extract of laves	H_2_O_2_- and CCl_4_-induced hepatotoxicity in goat liver	100 mg/kg	Oral	↓ the elevated AST, ALP, ALT, and protein	[278]

*P. foetida*	EtOH extract of leaves	CCl_4_-induced hepatic lesions and oxidative stress in rats	200 mg/kg	Oral	↓ the elevated LPO, GPT, GOT, ALP, and TB	[279]

*P. acidus*	EtOH and AQ extracts of leaves	APAP- and TAA-induced hepatic injuries in rats	200 and 400 mg/kg	Oral	↓ the elevated oxidative stress and serum AST, ALT, ALP, and TB	[280]

*P. emblica*	AQ extracts of fruits	APAP-induced hepatic damage in rats	100 and 200 mg/kg	Oral	Antioxidant properties were associated with its liver protective activity	[281]

*P. oleracea*	AQ extract of aerial parts	CCl4-induced hepatopathy in rats	50 mg/kg	Oral	↓ the elevated AST, ALT, ALP, TB, TP, and TC. It also protects drug-metabolizing enzymes	[282]

*P. indica*	Alcoholic leaf extract	CCl_4_-induced liver damage in rats	200 mg/kg	Oral	↓ the elevated GOT, GPT, and ALP	[283]

*P. nigrum*	AQ, EtOH, and CHCl_3_ extract of root	CCl_4_-induced rat liver injury	120 mg/kg	Oral	↓ the elevated ALT, AST, and MDA, as well as ↑ liver enzyme GSH	[284]

*R. vesicarius*	MeOH extract of the whole plant	CCl_4_-induced hepatotoxicity in the rat model	100 and 200 mg/kg	Oral	↑ liver enzyme SOD and CAT and ↓ the elevated SGPT, SGOT, ALP, MDA, and TB	[285]

*S. nigrum*	EtOH extract of leaves	In vitro free-radical-mediated DNA damage	NA	Cell culture	Prevented the free-radical-mediated oxidative degradation of DNA in the liver tissue debris to protect the liver	[286]

*S. torvum*	EtOH extract of fruits	Doxorubicin- (DOX-) induced hepatotoxicity in rats	100 and 300 mg/kg	Oral	↓ the elevated ALT and AST and ↑ liver enzyme SOD and CAT	[287]

*S. oleracea*	PE, EtOH, and AQ extract of seed	CCl_4_-induced hepatotoxicity in rats	100 *μ*g/mL	Oral	Restoration of biochemical and histological changes	[288]

*S. jambos*	EtOH extract of leaves	CCl_4_-induced liver damage in rat	300 mg/kg	Oral	↓ the elevated SGPT, SGOT, ALP, TB, TP, and liver weight	[289]

*T. purpurea*	EtOH extract of leaves	CCl_4_-induced liver damage in rat	100 mg/kg	Oral	↓ the elevated ALP, AST, ALT, and TB	[162]

*T. arjuna*	AQ extract of bark	INZ-induced liver toxicity in rat	200 mg/kg	Oral	↓ the elevated SGPT, SGOT, ALP, ACP, TB, and protein, as well as ↑ liver enzyme GSH and SOD	[88]

*T. bellirica*	AQ fraction of MeOH extract of fruit	CCl4-induced liver injury in rats and mice	50, 100, 200, and 400 mg/kg	Oral	↑ liver drug metabolizing enzyme and ↓ the elevated tranaminases, bilirubin, and LPO	[93]

*T. chebula*	EtOH extract of fruit	Liver toxicity induced by RIF, INZ, and PZA (in combination)	50, 100, and 200 mg/kg	Oral	↑ antioxidative and membrane-stabilizing activities, as well as ↓ the elevated SGPT, SGOT, ALP, and TB	[89]

*T. cordifolia*	Swaras and hima extract of fresh stems	APAP-induced hepatotoxicity in mice	200 mg/kg	Oral	↓ the elevated SGOT and ALP	[290]

*T*. *portulacastrum*	EtOH leaf extract	APAP- and TAA-induced liver toxicity in rats	100 and 200 mg/kg	Oral	↓ the elevated SGOT, SGPT, ALP, and TP	[291]
*T. dioica*	AQ and EtOH extract of aerial parts	Ferrous sulphate- (FeSO4-) induced liver injury in rats	100, 200, and 400 mg/kg	Oral	↓ the elevated AST, ALT, TB, and ALP and increased TP level	[292]

*W. chinensis*	Hot AQ extract of the whole plant	Acute hepatitis induced by CCl_4_, APAP in mice, and GNH_2_ in rats	300 mg/kg	Oral	↓ the elevated SGOT and SGPT	[293]
	EtOHc and AQ extract of the whole plant	CCl_4_-induced hepatotoxicity in rat	500 mg/kg	Oral	↓ the elevated SGOT, SGPT, ALP, and TB	[294]

**Table 3:** List of lead hepatoprotective compounds isolated form Bangladeshi traditional plants.

Plant name	Isolated compound	Test model	Dose	Route	Mechanism of hepatoprotective action	Ref.
*A. vera*	Polysaccharides	Alcohol-induced liver diseases in mice	10 mg/kg	IP	Reduced liver biomarkers via increasing lipolysis through upregulating hepatic expression of lipolytic genes AMPK-*α*2 and PPAR*α,* as well as reduced hepatic inflammation via downregulation of TLR-4 and MyD88 with upregulation of I*κ*B-*α*	[236]
	Aloe emodin	Myofibroblastic differentiation study in rat hepatic stellate cells	0.004–0.04 *μ*M/mL	Cell culture	Inhibition of stellate cell proliferation by reduced DNA synthesis and inhibition of type I collagen production and smooth muscle alpha-actin expression	[221]
		CCl_4_-induced hepatic injury in rats	185 *μ*M/kg	IP	Reduced hepatocyte death and inflammation through inhibition of TNF-*α* and LPO	[220]
	Aloin	Alcohol-induced liver injury in mice	24 and 72 *μ*M/kg	Oral	Attenuated lipid accumulation via inhibition of SREBP-1c regulate gene, as well as reduced hepatic inflammation through downregulation of TLR-4 and TNF-*α*	[223]
		TAA-induced hepatic retinopathy	120 and 240 *μ*M/kg	Oral	Suppressed retinal injury associated with liver toxicity through the normalization of Kir4.1 and aquaporin-4 channels	[224]

*A. spinosus*	Rutin	CCl_4_-induced hepatic damage in rats	NA	Oral	↓ the elevated level of transaminases, phosphatases, total protein, albumin and LPO as well as ↑ upregulation of antioxidant enzymes	[157]

*A. paniculata*	Andrographolide	EtOH-induced liver toxicity in mice	177–1427 *μ*M/kg	IP	Restored the elevated serum level of GOT, GPT, ACP, ALP, and LP	[175]
		Nonalcoholic high-fat-diet-induced fatty liver disease in rat	143 *μ*M/kg	Oral	Restored the elevated level of serum ALT, AST, and ALP, as well as normalized the hepatic architecture	[183]
		Palmitate-oleate-induced steatotic in HepG2 cells	12.5–50 *μ*M	Cell culture	Ameliorated hepatic steatosis and lipotoxicity via reduced lipid accumulation	
		APAP-induced liver damage in rat	2–34 *μ*M/kg	Oral	Increased viability of hepatocytes and ↓ the elevated SGOT, SGPT, and ALP in serum and isolated rat hepatocytes	[176]
		CCl_4_ and tert-butylhydroperoxide (t-BHP) intoxicated mice	286 *μ*M/kg	IP	↓ the elevatedMDA, SGPT, and ALP and ↑ liver GSH activity	[177]
		CCl_4_-induced liver toxicity in male mice	286 *μ*M/kg	IP	↓ the elevated SGPT, SGOP, ALP, TB, and TG, as well as protected drug-metabolizing enzyme	[178]
	Arabinogalactan proteins	EtOH-induced liver toxicity in mice	62–500 mg/kg	IP	Restored the elevated serum level of GOT, GPT, ACP, ALP, and LP	[175]
	Isoandrographolide	Nonalcoholic high-fat-diet-induced fatty liver disease in rat	143 *μ*M/kg	Oral	Restored the elevated level of serum ALT, AST, and ALP, as well as normalized the hepatic architecture	[183]
		Palmitate-oleate-induced steatotic in HepG2 cells	12.5–50 *μ*M	Cell culture	Reduced lipid accumulation and leakage of LDH and transaminases (ALT and AST)	
	3,19-Acetonylidene andrographolide	Nonalcoholic high-fat-diet-induced fatty liver disease in rat	128 *μ*M/kg	Oral	Restored the elevated level of serum ALT, AST, and ALP, as well as normalized the hepatic architecture	
		Palmitate-oleate-induced steatotic in HepG2 cells	12.5–50 *μ*M	Cell culture	Ameliorated hepatic steatosis and lipotoxicity via reduced lipid accumulation	
	Andrographiside	CCl_4_ and t-BHP intoxicated mice	195 *μ*M/kg	IP	↓ the elevated MDA, SGPT, and ALP and ↑ liver GSH activity	[177]
	Neoandrographolide	CCl_4_ and t-BHP intoxicated mice	208 *μ*M/kg	IP	↓ the elevated MDA, SGPT, and ALP and ↑ liver GSH activity	

*B. orellana*	Bixin	High-fat-diet-induced obese mice	127 *μ*M/kg	Oral	↓ all metabolic parameters including body weight, Lee's index, adiposity, CHT, TG, CHT/HDL-c, glucose, AST, and ALT	[234]

*C. cajan*	43 kD protein	TAA-induced liver toxicity in mice	2 mg/kg	IP	↓ the elevated SGPT, ALP, and LPO, as well as ↑ liver enzymes SOD, CAT, and GST	[295]

*C. tinctorius*	Kaempferol 3-O-rutinoside and kaempferol 3-O-glucoside	CCl4-induced oxidative liver injury in mice	336 and 672 *μ*M/kg	Oral	↓ the elevated AST, ALP, and MDA and ↑ liver enzyme GSH, SOD, and CAT, as well as normailized hepatocyte architecture	[158]

*C. fistula*	Catechin	STZ-induced hepatic injury in diabetic rats	69 *μ*M/kg	Oral	↓ the elevated AST, ALT, ALP, LDL, HDL, TC, and TG and normalized hepatic and renal cell damage	[159]

*C. longa*	Curcumin	HgCl_2_-induced liver toxicity in rats	217 *μ*M/kg	Oral	↓ the elevated ALP, LDH, TB, *γ*-GT, MDA, and TG, as well as ↑ the protective effect on drug-metabolizing CYP 2E1 enzymes	[123]
		Lindane-induced oxidative stress in male rats	272 and 544 *μ*M/kg	Oral	Decreases LPO and ↑ liver antioxidant enzyme SOD, GSH, CAT, GST), GPx, GR and NADPH quinine reductase (QR)	[203]
		Dimethylnitrosamine- (DMN-) induced liver cirrhosis in rats	272 *μ*M/kg	Oral	Restored the electrical conductivity and ↓ the elevated AST and ALT, as well as attenuated fibrosis and inflammatory response	[209]
		CCl_4_-induced hepatotoxicity in rats and mice	136 and 272 *μ*M/kg	Oral	↓ the elevated AST, ALT, ALP, and MDA and ↑ liver enzyme GSH and CAT, as well as normalized hepatic inflammatory lessions	[201]
		APAP-induced liver damage in mice	544 *μ*M/kg	Oral	↓ the elevated ALT, AST, and LPO, as well as ↑ liver enzyme SOD, CAT, and GPx	[202]
		Alfatoxin B1 (AFB1)-induced hepatotoxicity in rats	544 *μ*M/kg	Oral	↓ the elevated liver marker enzymes and LPO and ↑ liver enzyme GSH, SOD, CAT, and GPx	[205]
		AFB1-induced hepatotoxicity in rats	0.05% w/w with diet	Oral	Modulated drug-metabolizing enzyme and ↓ AFB(1)-N(7)-guanine adduct excretion in the urine, DNA adduct in the liver, and albumin adduct in the serum	[204]
		TAA-induced hepatic fibrosis in mice	814 *μ*M/kg	Oral	Inhibiting HSC activation and inflammatory responses and inducing apoptosis of damaged hepatocytes via upregulating p53 protein expression and downregulating Bcl-2 mRNA expression	[214]
		Hepatitis B (HBV)-transfection HepG2215 cell line	50–150 *µ*M	Cell culture	Inhibits HBV gene expression and DNA replication via downregulation of PGC-1*α*	[217]
	Sesquiterpene fraction: ar-turmerone, *α*-tumerone, and *β*-tumerone	D-galactosamine-induced liver injury in rats	0.5% w/w with diet	Oral	Suppressed the elevated LDB, ALT, and AST levels	[218]

*E. ribes*	Embelin	CCl_4_-induced peroxidative liver damage in rats	85 *µ*M/kg	Oral	Upregulatioon of liver antioxidant and cytochorme P450 enzymes, as well as ↓ the elevated AST, ALT, ALP, *γ*-GT, LPO, and LDH	[228]
		DENA-/PB-induced hepatocarcinogenesis in rats	170 *µ*M/kg	Oral	↓ hepatic hyper plastic nodules, body weight loss, and hepatic diagnostic markers	[226]
		DENA/PB-induced hepatocarcinogenesis in rats	170 *µ*M/kg	Oral	Upregulated the hepatic glutathione antioxidant defense, ↓ LPO, and minimized the histological alterations	[227]
		CCl_4_-induced liver toxicity in rats	170 and 240 *µ*M/kg	Oral	↓ the elevated SGPT, SGOT, ALP, *γ*-GT, GST, and lipid peroxidase, as well as ↑ liver glutathione and reduced cellular inflammation	[296]

*I. tinctora*	*trans*-Tetracos-15-enoic acid (TCA)	CCl_4_- and APAP-induced hepatotoxicity in rats and mice	34–273 *µ*M/kg	Oral	Accelerated regeneration of parenchymal cells and ↓ membrane fragility, LPO, and leakage of marker enzymes, as well as ↑ liver GSH	[232]

*L. inermis*	Gallic acid	CCl_4_-induced hepatotoxicity in rats	294 *µ*M/kg	IP	↓ the elevated ALT, AST, ALP, LDH, and ROS, as well as ↑ liver SOD, CAT, and GPx and normalized hepatocellular architecture	[170]

*M. charantea*	Cucurbitane-type triterpene glycosides	Antihepatic fibrosis activity against murine hepatic stellate cells (t-HSC/Cl-6) and antihepatoma activity in HepG2 and Hep3B cells	Upto 100 *µ*M	Cell culture	Inhibition the activation of t-HSC/Cl-6 cells and ↓ cytotoxicity of Hep3B and HepG2 cells	[197]
	Norcucurbitane-type triterpenoids	*t*-BHP-induced injury on HepG2 cells	5–10 *µ*M	Cell culture	↑ the viability of HepG2 cells	[198]

*O. basilicum*	Triterpene acid: betulinic, oleanolic, ursolic, alphitolic, 3-epimaslinic, and euscaphic acids	Iron ascorbate-stimulated lipid peroxidation in liver homogenate	0.1–5 mg/mL	Cell culture	↓ liver oxidative stress by inhibition of LPO	[187]

*P. olerace*	Portulene diterpene, lupeol, b-sitosterol, and daucosterol	CCl_4_-induced hepatic toxicity in rats	10–50 mg/kg	Oral	↓ the elevated level of SGOT, SGPT, and TB	[184]

*S. jambos*	Flavonoid fraction: myricetin, ellagic acid rahmnoside, quercetin 3-O-xylosyl-(1 ⟶ 2), rhamnoside, and rosmarinic acid	CCl_4_-induced liver toxicity in rats	200 mg/kg	Oral	↓ the elevated liver markers ALT, AST, TB, TC, TG, and MDA, as well as ↑ liver enzyme GSH and SOD	[167]
		Sodium arsenite-induced oxidative stress in HepG2 hepatocytes	50 *µ*gmL	Cell culture	↓ the ROS production via inhibition of p38 and its target MAPKAPK-2-activated signaling cascade	

*T. purpurea*	Flavonoid fraction: Coumarins, flavonoids, flavanones, and quercetin	CCl_4_-induced hepatotoxicity	100 mg/kg	Oral	↓ the elevated liver markers SGOT, SGPT, ALP, and bilirubin	[162]

*T. chebula*	Chebulic acid	t-BHP-induced oxidative stress in isolated rat hepatocytes	280 *µ*M/mL	Cell culture	↓ the ROS and cell cytotoxicity and the ratio of GSSH with GSH	[172]
		t-BHP-induced oxidative stress in HepG2 cells	0.4, 2, and 10 *µ*M	Cell culture	↓ the oxidative stress through controlling the activation of Nrf2 and its cytoprotective enzymes HO-1 and *γ*-GCL	[173]

## Data Availability

All relevant data are available within this manuscript.

## Abbreviation

AH: Aniline hydroxylase
AMPK-*α*2: AMP-activated protein kinase-*α*2
AND: Amidopyrine-N-demethylase
APAP: Paracetamol
ALD: Alcohol liver disease
ALP: Alkaline phosphatase
AQ: Aqueous
ARE: Antioxidant response element
AST: Aspartate aminotransferase
BHPs: Bangladeshi hepatoprotective plants
BHC: Hexachlorocyclohexane
t-BHP: tert-Butyl hydroperoxide
BSP: Bromosulphalein
CAT: Catalase
CCl_4_: Carbontetrachloride
COX-2: Cyclooxygenase-2
CYP2E1: Cytochrome P450-2E1
DEN: Diethyl nitrosamine
DENA: N-nitrosodiethylamine
DMN: Dimethylnitrosamine
EtOH: Ethanol
FeSO_4_: Ferrus sulphate
GA: Gallic acid
*γ*-GCL: Gamma-glutamate cysteine ligase
GNH_2_: D (+)-galactosamine
GSH: Glutathione
GPx: Glutathione peroxidase
GRD: Glutathione reductase
GST: Glutathione s-transferease
*γ* –GT: Gamma-glutamyl transferase
HAV: Hepatitis A virus
HBV: Hepatitis B virus
HCV: Hepatitis C virus
HEV: Hepatitis E virus
HgCl_2_: Mercuric chloride
HMGB1: High-mobility group protein box-1
HO-1: Heme oxygenase-1
HP: Hepatoprotective plant
t-HSC/Cl-6: Murine hepatic stellate cells
I*κ*B-*α*: Nuclear factor of kappa light polypeptide gene enhancer in B-cell inhibitor, alpha
IL-6: Interleukin -6
INZ: Isoniazid
iNOS: Nitric oxide synthase
JNK: c-Jun NH_2_-terminal kinase
Kep1: Kelch-like ECH-associated protein-1
LD: Lindane
LDH: Lactate dehydrogenase
LD_50_: Lethal dose 50%
LPO: Lipid peroxidation
MAPK: Mitogen-activated protein kinase
MAPKAPK-2: MAPK protein kinase-2
MDA: Malondialdehyde
MeOH: Methanol
MoA: Mechanisms of action
MyD88: Myeloid differentiation factor 88
NF-kB: Nuclear factor-kappa B
NOS: Reactive nitrogen species
NPSM: Natural product small molecules
NQO-1: NAD(P)H:quinone oxidoreductase-1
Nrf2: Nuclear factor erythroid-2-related factor-2
PB: Phenobarbital
PGC-1*α*: Peroxisome proliferator-activated receptor-gamma coactivator-1alpha
PPAR*α*: Peroxisome proliferator-activated receptor alpha
Prx-6: Peroxiredoxin-6
PT: Prothrombin time
PZA: Pyrazinamide
RdRp: RNA-dependent RNA polymerase
RIF: Rifampicin
ROS: Reactive oxygen species
SGOT: Serum glutamic oxaloacetic transaminase
SGPT: Serum glutamic pyruvic transaminase
*α*-SMA: Alpha-smooth muscle actin
sm-*α*: Smooth muscle *α*-actin
Smad4: Decapentaplegic homolog 4
SOD: Superoxide dismutase
SREBP-1c: Sterol regulatory element-binding protein-1c
TAA: Thiacetamide
TB: Total bilirubin
TC: Total cholesterol
TCA: *trans*-Tetracos-15-enoic acid
TG: Tryglyceride
TGF-*β*: Transforming growth factor-*β*1
TLR2: Toll-like receptor 2
TLR4: Toll-like receptor 4
TNF-*α*: Tumor necrosis factor-*α*
TP: Total protein
UGT: UDP-glucuronosyl transferase.
